# Detection of Different Hosts From a Distance Alters the Behaviour and Bioelectrical Activity of *Cuscuta racemosa*

**DOI:** 10.3389/fpls.2021.594195

**Published:** 2021-03-18

**Authors:** André Geremia Parise, Gabriela Niemeyer Reissig, Luis Felipe Basso, Luiz Gustavo Schultz Senko, Thiago Francisco de Carvalho Oliveira, Gabriel Ricardo Aguilera de Toledo, Arlan Silva Ferreira, Gustavo Maia Souza

**Affiliations:** ^1^Laboratory of Plant Cognition and Electrophysiology, Department of Botany, Institute of Biology, Federal University of Pelotas, Pelotas, Brazil; ^2^Department of Physics, Federal University of Pelotas, Pelotas, Brazil

**Keywords:** *Cuscuta*, parasitic plants, attention, 1/*f* noise, machine learning, plant cognition, plant-plant interaction, plant electrophysiology

## Abstract

In our study, we investigated some physiological and ecological aspects of the life of *Cuscuta racemosa* Mart. (Convolvulaceae) plants with the hypothesis that they recognise different hosts at a distance from them, and they change their survival strategy depending on what they detect. We also hypothesised that, as an attempt of prolonging their survival through photosynthesis, the synthesis of chlorophylls (a phenomenon not completely explained in these parasitic plants) would be increased if the plants don’t detect a host. We quantified the pigments related to photosynthesis in different treatments and employed techniques such as electrophysiological time series recording, analyses of the complexity of the obtained signals, and machine learning classification to test our hypotheses. The results demonstrate that the absence of a host increases the amounts of chlorophyll *a*, chlorophyll *b*, and β-carotene in these plants, and the content varied depending on the host presented. Besides, the electrical signalling of dodders changes according to the species of host perceived in patterns detectable by machine learning techniques, suggesting that they recognise from a distance different host species. Our results indicate that electrical signalling might underpin important processes such as foraging in plants. Finally, we found evidence for a likely process of attention in the dodders toward the host plants. This is probably to be the first empirical evidence for attention in plants and has important implications on plant cognition studies.

## Introduction

Dodder plants (Convolvulaceae: *Cuscuta* spp.) belong to a genus of holoparasitic plants distributed all over the world, except Antarctica (Birschwilks et al., 2006; Costea et al., 2011, 2015). These plants do not possess roots and their leaves are reduced to vestigial scales without photosynthetic function. Although heterotrophic, some species have retained the ability to photosynthesise (MacLeod, 1961; Ciferri and Poma, 1962, 1963; Machado and Zetsche, 1990; Hibberd et al., 1998). However, all the water, nutrients and the overwhelming majority of photoassimilates they need to survive come from their hosts (Jeschke et al., 1994; Hibberd et al., 1998).

Due to these characteristics, these heterotrophic plants share with herbivorous animals the need of locating their host plants and develop strategic behaviours to maximise their chances of survival and increase their fitness (Kelly, 1990, 1992; Mescher et al., 2009). Many dodder species have retained their ability to synthesise chlorophylls and even make photosynthesis, although in a very low rate (MacLeod, 1961; Ciferri and Poma, 1962, 1963; Pattee et al., 1965; Baccarini, 1966; Laudi, 1968; Machado and Zetsche, 1990; Choudhury and Sahu, 1999). Throughout more than a 100 years some authors have suggested that they retained this ability because it can be useful for extending their survival during their free life stage as a seedling or when they are separated from their hosts (Peirce, 1894; Pizzolongo, 1963; Pattee et al., 1965; Laudi, 1968; Lyshede, 1985).

Since the 19th century, it is known that dodders forage and make choices (von Mohl, 1827; Koch, 1874; Peirce, 1894; Kelly, 1990, 1992; Koch et al., 2004; Runyon et al., 2006; Wu et al., 2019), yet the mechanisms by which these behaviours emerge are still unclear. However, there are two likely mechanisms for host detection: volatile organic compounds (VOCs) emitted by the host plants, and/or by light cues (Albert et al., 2008).

Interestingly, when provided with options, dodders seem to choose what might be best for their survival. This phenomenon was first observed by von Mohl (1827) and, later, by Koch (1874). Koch (1874) used the word “capacity of choice” (*Wahlfähigkeit*) to describe this behaviour. According to him, seedlings of *Cuscuta lupuliformis* Krock. “show a certain, as yet unexplained, capacity of choice which physiological benefits are obvious” (Koch, 1874). Peirce (1894) supported the ability of dodders in choosing and, after a gap of almost 100 years, Kelly (1990, 1992) resumed the studies on decision-making in *Cuscuta*. It is noteworthy that Lyshede (1985) also have observed this phenomenon in *Cuscuta pedicellata* Ledeb.

Kelly (1990, 1992) claimed that the response of an organism toward a source of resources depends on the expected reward to be obtained, and that this gauging happens before the exploitation of these resources (Kelly, 1990). Then, Kelly (1990) demonstrated that *Cuscuta subinclusa* Durand and Hilg. is able to differentiate between diverse host species and that it invests energy and resources in the coiling around the host’s stem depending on the species of the host. The proportion of the investment is related to the proportion of the expected reward (Kelly, 1990). In a subsequent experiment, she demonstrated that *Cuscuta europaea* L. can detect different nutritional levels of its hosts, and it “rejects” hosts with poor nutritional quality after simply touching the host’s bark (Kelly, 1992). The hypothesis that dodders forage was supported by Koch et al. (2004), who performed experiments with *Cuscuta campestris* Yunck. for evaluating the foraging of the dodder when different host species were simultaneously present. They concluded that there is strong evidence that dodders forage for choosing the best hosts (Koch et al., 2004). Kelly’s (1990, 1992) and Koch et al.’s (2004) studies suggest that dodders have some “preuptake mechanism” for selecting potential hosts (Koch et al., 2004).

Runyon et al. (2006) have demonstrated that dodders forage and make choices based on the VOCs they perceive in the environment. The idea that dodders could show certain chemotropism was proposed by Bünning and Kautt (1956), but until then not confirmed. In Runyon et al.’s (2006) study, they demonstrated that seedlings of *Cuscuta pentagona* Engelm. were attracted by the VOCs emitted by potential hosts and used these cues for selecting and reaching them. Between wheat and tomato plants, the dodder mainly chose tomato. However, when wheat alone was presented to the dodder, the seedling grew toward it. The same was observed when extracted volatiles of both hosts were presented to the dodders (Runyon et al., 2006).

Another way by which dodders could detect and select among hosts is through light cues. Orr et al. (1996) demonstrated that *Cuscuta planiflora* Ten. shows phototropism toward low ratios of red/far-red light, even when they are under white light. Accordingly, Benvenuti et al. (2005) revealed that *C. campestris* can use red/far-red ratios as cues for selecting between hosts. Low ratios of red/far-red light were predictive of healthy leaves with abundant chlorophyll, and the dodders were strongly attracted by sugar beet leaves that transmitted this combination of wavelengths (Benvenuti et al., 2005).

These studies and others on *Cuscuta* show that these plants possess exquisite sensorial abilities to detect, locate, choose, and parasitise their hosts. Beyond, they indicate that both the species and characteristics of the hosts influence the dodders’ behaviour and predation strategies. However, most of these studies are behavioural and little is known about how the interaction from a distance of dodders with its hosts influence their physiology.

We have observed (Parise et al., in press) that excised twigs of *Cuscuta racemosa* Mart. accumulate green pigments within a few days. Then, acknowledging that (i) dodders detect hosts from a distance and change their behaviour accordingly, and assuming that (ii) dodders would synthesise photosynthetic pigments in order to prolong their lives in the absence of a host, we have hypothesised that the presence of a viable host near an excised twig of dodder would influence in its pigment content.

However, before this response to the hosts happen, other physiological processes must occur. A likely candidate for early detection of hosts is electrical signalling (Debono and Souza, 2019; Simmi et al., 2020). Electrical signals generated in the cells’ surface due to the detection of the host could rapidly spread to the entire plant and trigger other physiological processes.

Plants have a spontaneous, non-evoked, electrical signalling activity which is related to basic physiological processes (Fromm et al., 2013). Depending on the stimulus received from the environment, however, this basic electrical signalling can change. Numerous different kinds of electrical signals are produced for codifying the stimuli received and participate in the coordination of the responses to them. These signals range from the well-known action potentials to other less acknowledged such as system potentials (Fromm and Lautner, 2007; Zimmermann et al., 2009, 2016; Vodeneev et al., 2016). Electrical signals are of paramount importance to plants and they are related to a myriad of physiological processes, such as inducing transcription of genes, mounting defence responses, activating photosynthesis, synthesising hormones, etc. (de Toledo et al., 2019; Sukhov et al., 2019).

Electrical signalling is a hallmark of life itself. It manifests from the basic level of cell (or even organelles) to the level of an entire organism (De Loof, 2016). For referring to the totality of the electrical activity occurring in any organism or part of it during an amount of time, De Loof (2016) coined the term “electrome” inspired by the other “omic” sciences, such as genomics, transcriptomics, proteomics, and so on. Recently, Souza et al. (2017) brought to the plant science the term and some studies have been performed with plants, demonstrating that they have a lively and extremely active electrome that is related to many physiological states (Saraiva et al., 2017; Souza et al., 2017; Pereira et al., 2018; de Toledo et al., 2019; Simmi et al., 2020). It was demonstrated that the overall electrical activity of plants changes considerably depending on the stimulus the plant is receiving (Saraiva et al., 2017; Souza et al., 2017). By using automatic classification algorithms of machine learning, it is even possible to find patterns in the electrical response to many different stimuli, despite the particularity and individuality of each plant (Pereira et al., 2018; Simmi et al., 2020).

This tight connection of electrical signalling to environmental stimuli, and to the subsequent changes in other physiological processes (electrical signals precede many of them) led Debono and Souza (2019) to propose that the electrome is a pivotal interface that mediates the external environment with the internal. It would be a bioelectrical interface between the plant and the environment (Debono and Souza, 2019).

Here, we studied the species *Cuscuta racemosa* Mart. There are few studies on the physiology of this species and they are focused on the pharmacological properties of this plant (Ferraz et al., 2010, 2011; Sousa et al., 2012). Besides, as far as we know, no studies have examined the electrical signalling of a parasitic plant. Since dodders can cause huge crop losses worldwide (Bewick et al., 1988; Dawson, 1989; Parker, 1991; Costea and Tardif, 2006; Mishra, 2009; Goldwasser et al., 2012; Kaiser et al., 2015; Sarić-Krsmanović et al., 2015) and their control is extremely difficult after the attachment to their hosts, understanding how they detect and attach to them may be the key to protect crops against dodder infestation (Albert et al., 2008; Kaiser et al., 2015; Johnson et al., 2016).

Based on the ubiquity of electrical signalling in the plant kingdom and the literature about the foraging behaviour of *Cuscuta*, as well as the literature about the limited photosynthesis in this group of plants, we hypothesised that (1) *C. racemosa* would behave differently depending on the host it detects *from a distance*; (2) in order to maximise the use of its limited resources, it would synthesise more photosynthetic pigments (chlorophylls and carotenoids) only when it does not detect any host in its vicinity; (3) different hosts would elicit different concentrations of pigments depending on their viability to the dodder; (4) the detection of hosts from a distance would alter the electrome dynamics of *C. racemosa;* and (5) this alteration is different depending on the identity of the host detected. Furthermore, there is a pattern recognisable by machine learning techniques in the electrome of the dodders presented to different hosts.

## Materials and Methods

### Plant Material

In the spring of 2019, a twig of *C. racemosa* was collected from a public flowerbed in central Pelotas, RS, Brazil. It was brought to the Federal University of Pelotas and cultivated in greenhouse conditions (mean temperature of 28.5°C ± 12.9, natural sunlight) on basil plants (*Ocimum* sp.) which consisted in the stock of dodders used in this experiment. The basils were planted in common plant substrate and twice a week irrigated with approximately 200 mL of Hoagland and Arnon solution (Hoagland and Arnon, 1950).

### Basic Experimental Setup

**FIGURE 1:**
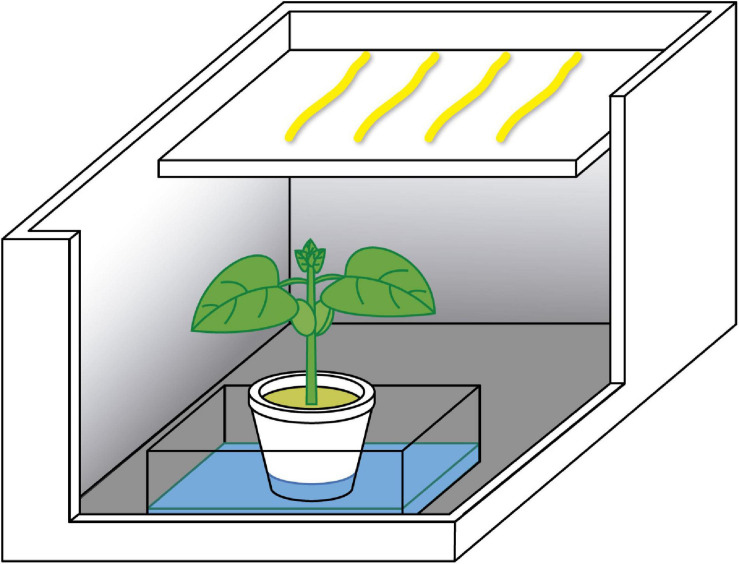
Schematic representation of the experimental setup. The dodder twigs (yellow) were placed on the shelf, and the host (bean plant, wheat plant, or wood sticks), inside the pit.

All the experiments carried out in this study were variations from the same basic model. The experimental setup consisted of a small shelf of polystyrene inserted in boxes with internal dimensions of 20.0 × 25.0 × 17.0 cm. The shelf was placed in one of the box’s extremity 5.0 cm deep inside the box and measured 12.0 × 17.0 cm (Figure 1).

It created a pit in the box, under the shelf, where the pot with the treatment was placed (a pot with a bean or wheat plant, sticks, or nothing/control). Four twigs of dodder were placed on the shelf, approximately in parallel among them and with the longest sides of the box. This setting was chosen for preventing the walls of the boxes from casting shade on the lateral dodders and not on the central ones, thus potentially interfering on the results. Besides, since different hosts have different shapes and chemical composition, the dodders were placed above them for minimising the interference of different light qualities transmitted or reflected by them on the pigment content. With this setting, the amount of light arriving to the dodders was the same. The apex of each twig was about 8.0–12.0 cm away from the host. In all the experiments, the polystyrene box was closed with a transparent polyvinyl chloride (PVC) film. Variations of this design were applied depending on the assay.

To test our hypotheses, experiments were carried out by presenting to the dodder plants different kinds of hosts (viable living, unviable living, and unviable dead hosts), as follows:

#### Viable Living Host (Bean Plant)

Bean plants (*Phaseolus vulgaris* L. cv. BRS-Expedito) were used as viable living hosts because dodders successfully parasitise these plants. They were sowed in Gerbox^®^ boxes on Germitest^®^ paper moistened with distilled water and kept under artificial lighting (a row of four white LEDs, 50 W, approx. 5,000 lm placed 70.0 cm above), photoperiod of 12.0 h and constant temperature of 25°C ± 2. About 4 days after sowing the seedlings were transferred to polystyrene pots of 120.0 mL filled with 150.0 g of sand. Then, they were brought to a greenhouse and daily irrigated with Hoagland and Arnon solution at half concentration (Hoagland and Arnon, 1950). When the plants were in the stage V2 of development, with the first trifoliolate leaf developing (de Oliveira et al., 2018), they were used in the experiments.

#### Unviable Living Host (Wheat Plant)

Wheat (*Triticum aestivum* L. cv. BRS-Parrudo) was chosen as the unviable living host, for it is known that dodders cannot parasitise these plants (Costea and Tardif, 2006; Albert et al., 2008). Indeed, if another host is offered to the dodders, they avoid wheat (Runyon et al., 2006). The same process for sowing and planting applied to the beans was applied to the wheat plants. They were used in the experiments when the third leaf was emerging after germination.

#### Unviable Dead Host (Wood Sticks)

For the unviable dead host, three bamboo sticks were vertically placed in a polystyrene pot with 120.0 mL of volume and filled with 150.0 g of sand. These were the same conditions as the pots with the beans and wheat.

### Experiment 1: Influence of Different Hosts on the Growth and Pigment Content of *C. racemosa*

This assay was designed to test hypotheses 1–3. Dodder twigs were collected from the *C. racemosa* stock and trimmed for all of them have 10.0 cm of length, measured from the basis of the node. Normally, these nodes present one main stem, one secondary stem, and one bud. The stems were excised and the buds were left. The node was left in the apical extremity of the twig. All the twigs used in this assay had masses ranging from 100.0 to 130.0 mg. Before being placed in the box, the length of the buds was measured.

The twigs were placed in the box’s shelf, in parallel and approximately equidistant between them (Figure 1). The pot containing the treatment (bean, wheat, sticks, or nothing) was put inside the box’s pit. Each pot containing the treatment was inside a Gerbox^®^ opened. Before the polystyrene box being closed with the PVC film, the host plants in each treatment were irrigated with 50.0 mL of Hoagland and Arnon nutrient solution (Hoagland and Arnon, 1950). For keeping humidity in the air of all the boxes, an equivalent amount of Hoagland and Arnon solution was poured inside the Gerbox^®^ boxes of the treatments without living plants.

The polystyrene boxes were closed with the PVC film and left in the laboratory’s growing room for 1 week, under artificial lighting (a row of four white LEDs, 50 W, approx. 5,000 lm placed 50.0 cm above), 12.0 h photoperiod, and constant mean temperature of 25.0°C ± 2. After this period, the twigs were taken from the boxes. Their fresh masses and the length of the new shoot were measured. Then, the content of chlorophyll *a*, chlorophyll *b*, lycopene, and β-carotene was quantified (modified from Nagata and Yamashita, 1992). By the end of the test, some of the new shoots touched the host plant but did not coil nor set haustoria.

### Experiment 2: Influence From a Distance of Different Hosts on the Electrome of *C. racemosa*

With Experiment 1, we observed that the content of pigments in the dodders change depending on the host they are perceiving. It suggests that the dodder’s perception of the host, from a distance, influences its internal physiology. Experiment 2 was designed to go deeper into the investigation of this perception and test hypotheses 4 and 5. For verifying whether the dodder can distinguish between hosts at the level of electrome (i.e., the sum of all its electrical activity, from cell to whole plant—or part of it), the treatment consisted in presenting to the dodders either a bean plant or a wheat plant. The control was made by measuring the electrome before the presentation to the hosts.

The experimental set was brought to a Faraday cage in the laboratory. It was used the electrophytography (EPG) technique to observe the dodders’ electrome dynamics (Gensler, 1974; de Toledo et al., 2019). The bioelectrical data acquisition was made with the system Biopac Student Lab (BIOPAC Systems^®^, Goleta, CA, United States), model MP36 with four channels with high input impedance (10 GΩ). Signals were collected with a sampling rate of *f*_*s*_ = 62.5 Hz amplified with a gain of 1,000-fold. The protocol used was ECG-AHA (0.05–100 Hz) with a notch frequency of 60.0 Hz for minimising the influence of the electrical network. No open electrode was left because it was well described in previous studies (Saraiva et al., 2017; Simmi et al., 2020). Open electrode voltage variation has a typical Gaussian noise with a lower amplitude than the plant signal baseline (Saraiva et al., 2017). Two needle electrodes (model EL-452, BIOPAC Systems^®^, Goleta, CA, United States) were inserted in the twigs, being one electrode immediately under the node and the other one inserted ca. 1.0 cm more basally. The boxes were closed with the PVC plastic film and let in the laboratory overnight for the acclimation of the plants. The experimental sets were kept under a white LED light (100 W, approx. 10.000 lm) with a photoperiod of 12.0 h and constant mean temperature of 25°C ± 1.

The experimental setup was basically the same described in Experiment 1. However, here, for allowing the insertion of the electrodes, the twigs had the highest masses as possible (mean 0.172 mg ± 0.042). Furthermore, the buds were longer than in Experiment 1 (mean 0.6 cm ± 0.3) to facilitate the perception of chemical and light cues.

After acclimation, the electrome of the dodders was recorded for 2.0 h before the test in the absence of any host. Then, the box was opened and a host was placed inside the pit. Immediately after, the box was closed again and the electrome recorded for 2.0 h more.

### Analyses

#### Growth Analyses

Initial and final fresh mass were measured with a semi-analytical balance. The shoot length was measured with a ruler. Since the length of the bud was variable, the initial length of the buds was subtracted from the final length in an attempt to normalise the growth of the shoots.

#### Pigment Analyses

For quantifying the pigment content, the modified protocol of Nagata and Yamashita (1992) was used. The dodders were macerated in hexane and acetone in a proportion of 2:3. Then, they were vortexed for 30.0 s. The supernatant was transferred to quartz cuvettes and the absorbances were measured in a spectrophotometer. The results were expressed in mg 100 g^–1^ of fresh mass. Each box contained four dodders (i.e., four observations per treatment). The mean value for all the treatments was calculated.

#### Electrophysiological Analyses

We analysed time series of micro-voltage variations as Δ*𝕍* = {Δ*V*_1_,Δ*V*_2_,…,Δ*V*_*N*_} obtained from the EPG, in which ΔV_*i*_ is the difference of potential between the electrodes inserted in the dodder’s twigs, and *N* is the length of the time series. This length is derived from a sample of 2 h (7,200 s) of data acquisition, and using *f*_*s*_ = 62.5 Hz, is *N* = 450,000 points. Each time series was analysed by the following techniques:

##### Visual analyses

Firstly, the EPG time series were submitted to visual analyses for a preliminary search for patterns or alterations in the time series. Although it is descriptive and susceptible to subjectivity, it allows a first analysis of the behaviour of the time series, as well as some comparisons between them. For example, the higher presence of spikes of voltage variation.

##### Fast Fourier transform

It demonstrates the frequency with which waves of ΔV with different amplitudes occur. It is important because, in a time series, different waves and amplitudes are overlapped, which hinders a visual verification of the dominant frequencies. The fast Fourier transform (FFT) decomposes the amplitude of waves in the spectrum of frequencies, evidencing which are the dominant frequencies of ΔV. For example, the alpha, beta, gamma, delta and theta waves of the brain are detected through this technique. In the brain, one or other of these waves will be more frequent depending on what it is experiencing (Michel et al., 1992; Nunez and Srinivasan, 2006).

##### Wavelet transform

This technique assembles both what time series and FFT demonstrate in the same graph. It evidences the occurrence of dominant frequencies and its amplitudes through time. This analysis is used in biological research for identifying the dominant frequencies in a time series because it shows, exactly, when these frequencies occurred (Akin, 2002; Adeli et al., 2003). However, this transform compromises the resolution of the analysis because when the resolution of frequency increases, the resolution of time decreases, and vice versa. For compensating for this loss of resolution in the domain of frequencies, it is common its employment together with the FFT (Whitcher et al., 2005; Hramov et al., 2015).

##### Mean of voltage variation

Despite being a rather simplistic measure, the mean of ΔV might offer general information about the electrome’s behaviour. For example, if it becomes more positive or negative after a stimulus, it may suggest a polarisation of the signals. Besides, in general, an increase in the mean suggests an increase in the occurrence of spikes with higher amplitude (de Toledo et al., 2019). In this work, the mean was calculated from the 450,000 sampling points obtained with the protocol adopted.

##### Histograms

It quantifies with which frequency a variable occurs within an interval of values. In this case, the variable is the micro-voltage variations (ΔV). When the noise is random, like the one obtained with an open electrode, the histogram that represents the ΔV events shows a typically Gaussian curve. The histograms generated from the time series of the electrome of plants usually have longer tails (for rare events like spikes with high amplitudes occur more often; de Toledo, 2019). From the histogram, we quantified some aspects of the distribution of the ΔV events such as the histogram’s asymmetry and kurtosis. Since histograms alone do not bring much information, we did not show them here.

##### Dispersion measures (standard deviation, asymmetry, and kurtosis)

These measures verify how the values disperse around the mean. In a general fashion, rare events tend to increase the standard deviation, making the histogram more asymmetric and decrease the kurtosis. It happens because the number of data points is limited (450,000). Therefore, if there is a dispersion away from the mean, fewer points will “remain” around the mean and it will cause a flattening of the histogram (decreased kurtosis). The dispersion can occur to the right or left side of the histogram, causing an asymmetry. In these cases, it is said that the asymmetry is positive or negative, respectively. Rare events such as spikes of ΔV concentrate in the tails of the histogram and the increase in the frequency of these events causes a decrease in the kurtosis and, frequently, an increase in the asymmetry (de Toledo, 2019). The mathematical description of these measures can be found in the Supplementary Material.

##### Autocorrelation function

This function shows how much two events separated in time are correlated, which enables the verification of patterns in the repetition of events through time. The greater the autocorrelation, the greater is the time interval between one event and another related to it. Thus, it indicates the existence of events of long duration or a greater persistence of the signals. In short, it measures how much an event of ΔV influences other events through time in the time series.

##### Probability density function

This function demonstrates the probability of a variable to occur in a certain point of the histogram through a regression of the distribution values. From the probability density function (PDF), it is possible to verify if there is a function that can describe the distribution of variables. Some studies in plant electrophysiology that used this analysis showed that a power law is the function that best describes the data obtained (e.g., Saraiva et al., 2017; Souza et al., 2017; Simmi et al., 2020). It indicates, for example, that there is no typical frequency, scale or amplitude for the ΔV events. The function of power law can be identified by the exponent μ of the equation PDF = *f*(|Δ*V*|)^∼^ |Δ*V*|^−μ^. When 1 < μ < 3, the function usually describes a distribution of values related to scale invariance, i.e., no characteristic size. For mathematical details of this analysis, see Supplementary Material.

##### Power spectral density function

This function shows how the spectral energy (the power of the waves) is distributed per unit of time (frequency). The power spectral density function (PSD) decays with a frequency *f*, generally following an equation that can be described as a power law such as PSD = 1/*f*^β^. Typically, the value of the exponent β varies between 0 and 3. The name of a colour is symbolically attributed to the respective kind of noise, depending on the value of the exponent β. For β = 0, it is said *white noise*, in reference to white light, in which all the frequencies have the same energy and are equally mixed. There is no linear correlation in the signals. For β = 1, it is said *pink noise*. This kind of noise is particularly frequent in high-complexity time series and indicate long-range correlation, scale-invariance and self-organised criticality (SOC). When the values of the exponent β lies between 0.5 and 1.5, it is called as reddened noise, which also present long-range correlation, though less complex than in pink noise (Cuddington and Yodzis, 1999). When β = 2, it is said *brown noise*, for it describes a pattern in the time series similar to Brownian moment, which has short-range correlation. Finally, when β = 3, it is said *black noise*, which is characterised by higher regularity and more short-range correlation when compared with β = 2 (Simmi et al., 2020).

A remarkable characteristic of living systems is the SOC. It indicates, bluntly put, that a dynamic system is in the imminence of changing its state (Bak, 1996). Generally, when a system is operating at this point, it is possible to observe interesting phenomena, such as pink noise, phenomena that occur without a characteristic scale of occurrence (i.e., scale invariance, fractality), and phenomena that occur following a power law (de Toledo, 2019). Therefore, the PSD is a good tool for investigating the complexity of electrical signals in time series.

##### Approximate entropy and multiscale sample entropy

These analyses provide information about the level of organisation of the time series. Higher values of Approximate entropy (*ApEn*) indicate the existence of more irregular dynamics (higher complexity), while lower values indicate that the dynamic is more regular and deterministic (Pincus, 1991, 1995). Deterministic processes have value *ApEn* = 0. Measurements from *ApEn* and *ApEn(s)* were developed to evaluate the level of complexity of real-world time series’ dynamics in terms of regularity and irregularity—or predictability and unpredictability. This method is commonly used in physiological and electrophysiological research and even in medicine (Costa et al., 2005). Despite being a robust method, *ApEn* measurements in a single scale may not be very accurate. This problem is overcome by the measurement of the *ApEn* in many scales *s*, which is the Multiscale Sample Entropy *ApEn(s)*. With *s* > > 1, for stochastic processes (*white noise*), the elements of the new time series Δ*𝕍*(s) converge to approximately equal values (single point in the phase space) and the entropy decreases, as shown in the Supplementary Figure 1B. When the process is of low complexity, increasing the size of the scale *s*, the elements never converge to a single value and entropy also increases (Supplementary Figure 1D). This increase is associated with always having new information on any scale *s*.

#### Machine Learning Analyses

For the machine learning analyses, in order to test hypothesis 4, we compared the electrome of the dodders before they were presented to their respective hosts with the electrome of the same plants after being presented to their hosts (dodder + bean *before* vs dodder + bean *after*, and dodder + wheat *before* vs dodder + wheat *after*). Then, for testing hypothesis 5 we compared the treatments between them, i.e., dodder + bean *before* vs dodder + wheat *before*, and dodder + bean *after* vs. dodder + wheat *after*.

All the time series were divided into 10 interchangeable parts between them with a lag of 30%, meaning that each part overlaps the other. Then, the FFT, Wavelet Transform, and the PSD were calculated. From these measures, we calculated the mean, maximum and minimum value, variance, skewness and kurtosis. Finally, the principal component analysis (PCA) was calculated with the features of the FFT, PSD and wavelet in order to obtain three features: PCA1, PCA2, and PCA3. In the end, we had a total of four features: PCA1, PCA2, PCA3, and entropy. These were the features used as input for the machine learning. For doing the graphs presented in the figures, we used PCA1 and entropy as coordinates.

Hyperparameters help to adjust the data to the machine learning technique. Despite the existence of discovery techniques for some of these hyperparameters, most are optimised only with trial and error. One of the main coexisting hyperparameters in every machine learning model is the division of its data group into training and testing. For obtaining a cohesive result and avoid overfitting or underfitting, we used the StratifiedKFold method, which separates the data, and the cross_validate method for the cross verification (Jabbar and Khan, 2014; Bronshtein, 2017). Then, we obtained a mean result of accuracy for each model, considering the different configurations of training and test groups and then eliminating the possibility of chance interference in our results.

We run 50 different data distribution of training and test for each treatment (CB-before, CB-after, and CW-before, etc.). Each round resulted in one different accuracy. Then, we calculated the arithmetic mean of all the 50 accuracies obtained and assumed as error the standard deviation. Beyond the accuracy, given in percentage, the standard deviation is given as a margin of error for each percentile. We analysed the result of the two best results obtained. The programming language used was Python, and the machine learning models were obtained from the open library Scikit-Learn (2020). The machine learning models used were:

##### DecisionTreeClassifier

It uses a decision tree as a predictive model for obtaining information about an item, represented in the branches, and the conclusions about the value of the target, which is represented in the leaves. In the decision analysis, a decision tree can be used to represent visually and explicitly the decisions and decision-making (Breiman et al., 1984).

##### SVC

The SVC (Support Vector Classification) derives from the SVM (Support Vector Machine) and is a model of supervised associated learning (Hsu et al., 2003). It uses analysis for classification and regression. An SVM training algorithm builds a model which attributes new examples to one or another category, which makes it a linear non-probabilistic binary classifier. An SVM model is a representation of the examples as points in the space, mapped for the examples of the separated categories to be divided by a blank gap the wider as possible. New examples are mapped in the same space and it is predicted that they belong to one or another category based in the side of the gap that they fall.

##### LinearSVC

It is similar to the SVC, but has more flexibility in the choice of penalty functions and losses. It must be dimensioned for a large number of samples. This class bears dense and sparse inputs (Ho and Lin, 2012).

##### GaussianProcessClassifier

It implements Gaussian processes (GPs) for regressions. GPs are a generic method of supervised learning. It was projected for solving problems of regression and probabilistic classification (Gibbs and MacKay, 2000).

##### KNeighborsClassifier

For pattern recognition, the nearest k-neighbours algorithm (k-NN) is a parametric method used for regression and classification. In both cases, the input consists of the k examples of training nearest in the space (Pandya, 2016).

##### RandomForestClassifier

It is a method of conjoint learning for classification and regression which operate building many decision trees during training, and generate the class of individual trees. The decision forests correct the trend of the trees in adjusting to their training dataset (Breiman, 2001; Fraiwan et al., 2012).

##### GaussianNB

Bayes naïve classifiers are a family of simple “probabilistic classifiers” based on the Bayes theorem. This method has a major facility in solving the problem of judging the classes as belonging to one category or another (Rasmussen and Williams, 2006).

##### Control

As a control, we used the DummyClassifier method, which uses different not intelligent strategies for classifying data (Engemann et al., 2018). Therefore, we have a basis for comparison. The model which obtains an accuracy close to the Dummy’s shall be considered as not suited for the dataset used. After some tests, the best Dummy model was the dummy_stratified, and therefore we used it as the control for all the analyses.

Finally, for further visualise the different patterns in the time series analysed, we made a scatter plot for the following categories: *Cuscuta* + bean before (CB-before) and CB-after, *Cuscuta* + wheat before (CW-before) and CW-after, CB-before and CW-before, and CB-after and CW-after. Two features of the features set were used for training the machine learning as coordinates.

### Experimental Design and Statistical Analyses

For Experiment 1, five repetitions were carried out, totalling 20 observations (four twigs of dodder per box). The experimental design was completely randomised and the data was analysed with ANOVA. When F was significant the treatments were compared with Tukey test (*p* ≤ 0.05).

For Experiment 2, six repetitions with four twigs of dodder per box were made, totalling 24 observations (24 time series before and 24 after the stimulus) per treatment. Due to a problem in one channel during the recording of the data, one observation for each treatment was discarded. Then, the total of observations analysed in this work was *n* = 23. Descriptive and quantitative analyses of the time series were made, as described in the previous section.

The mean of the values obtained before and after each stimulus (dependent variables) was compared by the paired *t*-test (*p* ≤ 0.05). When the data did not show normal distribution, the Wilcoxon Signed-Rank test (*p* ≤ 0.05) was used. For verifying whether the response of the dodders was different for each treatment (independent variables), the mean of the values before and after each stimulus was compared by Student’s *t*-test (*p* ≤ 0.05). When the data did not show normal distribution, the Mann-Whitney *U* test (*p* ≤ 0.05) was used.

## Results

### Experiment 1: Influence of Different Hosts on Growth and Pigment Content in *C. racemosa*

The results for the growth analyses of the dodder’s shoot in the presence of different kinds of hosts are presented in Table 1. There was no significant difference (*p* ≤ 0.05) in any of the growth parameters, except for the dodders presented to the wheat, in which the shoot was significantly (*p* ≤ 0.05) shorter than the shoot of the dodders presented to the bean and the control treatment. Nevertheless, there was no difference in the length of the shoot between the dodders presented to wheat and the sticks.

**TABLE 1:** Initial fresh mass (IFM), final fresh mass (FFM), and shoot length (SL) of the twigs of *C. racemosa* presented to different hosts.

	**Control**	***C.* + bean**	***C.* + sticks**	***C.* + wheat**
IFM (g)	0.116 ± 0.004 A	0.116 ± 0.006 A	0.113 ± 0.004 A	0.119 ± 0.005 A
FFM (g)	0.078 ± 0.009 A	0.078 ± 0.011 A	0.078 ± 0.009 A	0.078 ± 0.007 A
SL (cm)	13.18 ± 0.601 A	13.150 ± 0.717 A	12.670 ± 0.884 AB	12.503 ± 0.700 B

*Values represent the mean + standard deviation (*n* = 20). Means followed by the same capital letter, in the line, did not differ significantly (*p* ≤ 0.05) by the Tukey test. *C.* + bean: *Cuscuta* + bean; *C.* + sticks: *Cuscuta* + sticks; *C.* + wheat: *Cuscuta* + wheat; and Control: *Cuscuta* presented to no host.*

The values for the content of pigments in the dodders are shown in Table 2. The control treatment, with no host, was the one which led to the highest accumulation of chlorophyll *a*, *b*, and β-carotene. There was no significant difference (*p* ≤ 0.05) in the content of lycopene between the treatments.

**TABLE 2:** Chlorophyll *a*, chlorophyll *b*, lycopene, and β-carotene content in dodders presented to different hosts.

	**Control**	***C.* + bean**	***C.* + stick**	***C.* + wheat**
Chlorophyll *a*	6.303 ± 0.495 A	5.184 ± 0,515 B	5.515 ± 0.692 B	2.555 ± 0.296 C
Chlorophyll *b*	2.185 ± 0.257 A	1.829 ± 0.201 B	1.809 ± 0.290 B	0.849 ± 0.051 C
Lycopene	0.617 ± 0.187 A	0.494 ± 0.185 A	0.566 ± 0.196 A	0.537 ± 0.041 A
β-carotene	12.446 ± 0.659 A	11.563 ± 0.611 B	11.647 ± 0.444 B	7.11 ± 0.356 C

*Values represent the mean + standard deviation (*n* = 20). Means followed by the same capital letter did not differ significantly (*p* ≤ 0.05) by the Tukey test. *C.* + bean: *Cuscuta* + bean; *C.* + sticks: *Cuscuta* + sticks; *C.* + wheat: *Cuscuta* + wheat; and Control: *Cuscuta* presented to no host. All the values are expressed in mg 100 g^–1^.*

### Experiment 2: Influence From a Distance of Different Hosts on the Electrome of *C. racemosa*

#### Electrophysiological Analyses

**FIGURE 2:**
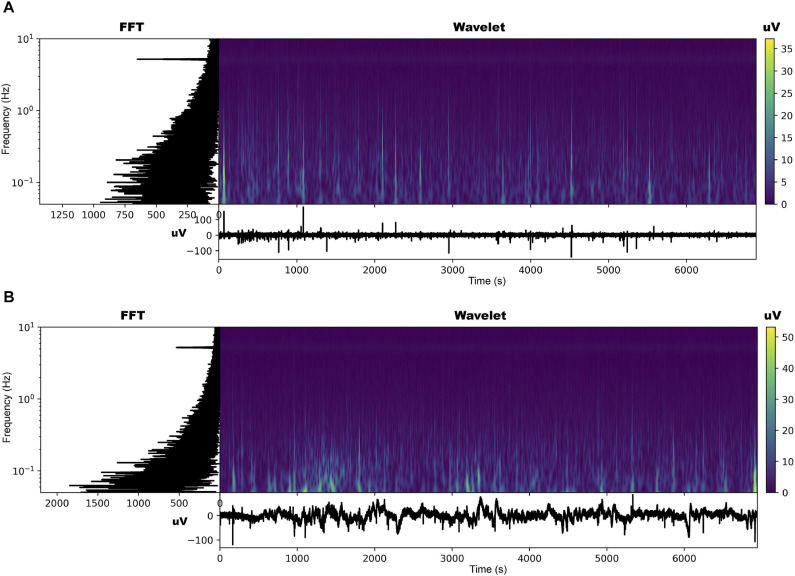
Time series, fast Fourier transform in log-linear scale, and wavelet transform for the dodder’s electrome before **(A)** and after **(B)** being presented to the bean plant.

**FIGURE 3:**
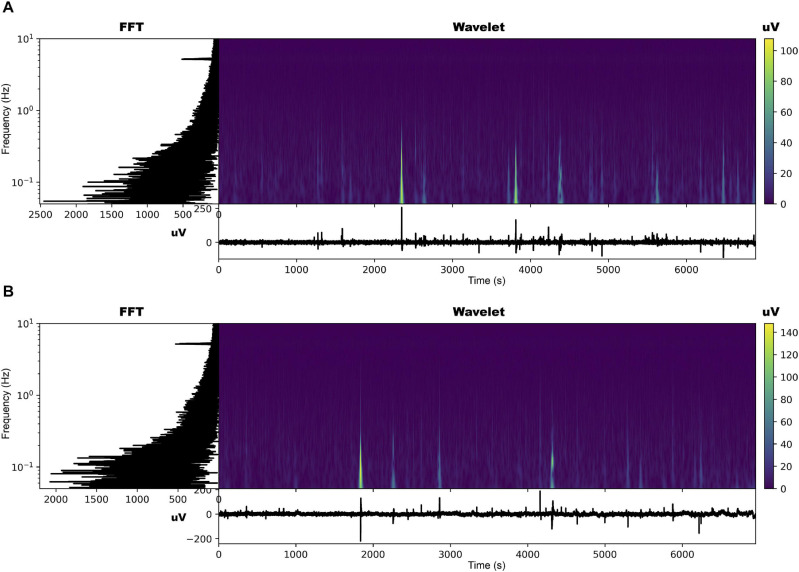
Time series, fast Fourier transform in log-linear scale, and wavelet transform for the dodder’s electrome before **(A)** and after **(B)** being presented to the wheat plant.

The EPG of the dodders presented to both hosts changed considerably, especially in those presented to the beans. The most noticeable change was the appearance of an undulating pattern in the time series, which oscillated around the mean in the form of long waves of voltage variation (Figures 2, 3 and Supplementary Figures 2, 3). These waves appear with more or less intensity in 20 out of 23 series analysed for the beans (86.9% of the observations). For the wheat, they appeared in 14 of the 23 time series analysed (60.7% of the observations).

The FFT analyses showed that, in both cases, in general, there was a decrease in the values of the dominant frequencies accompanied by an increase in their amplitude after the presentation to the hosts (Figures 2, 3 and Supplementary Figures 2, 3). What was observed in the time series and by the FFT analysis also is confirmed by the wavelet transform, in which lower frequencies with higher amplitudes appeared more frequently through the time in the presence of viable hosts. The constant frequency of 5.0 Hz observable in all the FFTs is mostly an artefact caused by the intrinsic and extrinsic noise of the experimental system (Kay, 2012).

The values obtained for the mean of the time series Δ*𝕍* = {Δ*V*_*i*_,*i* = 1,…,*N*}, the skewness (α), kurtosis (κ), the average correlation time *L*, the exponent β of the PSD, the exponent μ of the PDF and the *ApEn*(*s = 1*) are presented in Table 3. There was a significant increase (*p* ≤ 0.05) in the average values of the voltage variation after presenting the dodders to the hosts, which raised from −148.0 to 457.0 μV in the dodders presented to bean, and from 138.0 to 697.0 μV in the ones presented to wheat. There were no significant changes (*p* ≤ 0.05) in the skewness and kurtosis in both cases.

**TABLE 3:** Evaluated parameters of the electrical signal time series acquired in the experiment 2.

	**Treatments**			
	**Wheat**		**Bean**	
	**Before**	**After**	**Before**	**After**
Asymmetry^1^	−0.5062 ± 11.3357	−0.9695 ± 9.0661	−2.3578 ± 12.6820	0.4750 ± 11.4266
*p*-value	= −0.875		=0.311	
Kurtosis^2^	172.7551	159.3833	165.8386	53.7678
*p*-value	=0.659		=0.354	
ΔμV^3^	138.0	697.0**	−148.0	457.0**
*p*-value	=0.002		=0.009	
Average correlation time *L*^4^	36.50	127.19**	36.03	196.920**
*p*-value	≤ 0.001		≤ 0.001	
ApEn^5^	1.6058**	1.1835	1.5678**	0.9040
*p*-value	≤ 0.001		≤ 0.001	
PSD^6^	−1.2794 ± 0.2958	−1.4142* ± 0.2515	−1.1871 ± 0.2318	−1.2418 ± 0.2632
*p*-value	=0.001		=0.323	
PDF^7^	−4.7085 ± 1.2900	−4.5678 ± 1.2740	−4.8653 ± 1.2337	−4.5576 ± 0.9237
*p*-value	=0.764		=0.604	

Means followed by * differ significantly according to paired *t*-test (*p* ≤ 0.05). Values represent the mean ± SD. The Wilcoxon Signed Rank Test (*p* ≤ 0.05) was used for data that did not show a normal distribution. Medians followed by ** differ significantly. The values represent the medians. Before: without treatment; After: with treatment.^1^(*n* = 23). ^2^(*n* = 23). ^3^Voltage variation (*n* = 23). ^4^(*n* = 23). ^5^Approximate entropy (*n* = 23). ^6^Exponent β of the Power Spectral Density (*n* = 23). ^7^Probability density function (*n = 11*).

The analysis of the voltage variation events distributed on the PDF [*f*(|Δ*V*|)∼1/|Δ*V*|^−μ^] demonstrated that a power law is the function that suits to most of the cases for tail the PDF (11 time series for dodders presented to the beans, and 15 for dodders presented to the wheat). The other time series did not fit into any known PDF. In average, there was no difference in the values of the PDF exponent before and after presentation to the dodders.

After being presented to the hosts, the autocorrelation function (correlation time *L*) increased dramatically in both cases, bouncing from 37.139 ± 2.757 to 179.273 ± 13.028 in the bean treatment and from 42.739 ± 5.353 to 117.086 ± 16.445 in the wheat treatment.

The values of the exponent β of the Power Spectral Density (PSD ∼ 1/*f*^β^) was β = 1.19 ± 0.05 before and β = 1.24 ± 0.05 after the presentation to the host in the bean treatment, but this difference was not significant (*p* ≥ 0.05). However, in the wheat treatment, the values significantly increased (*p* ≤ 0.05) from 1.28 ± 0.3 to 1.41 ± 0.3 after the presentation to the wheat. Since all the values are close to β = 1, it can be described as a signal with pink-like noise, meaning that the signals obtained have long-range correlation, are highly complex and organised, and are scale-invariant, having information in all the levels of organisation (He et al., 2010; He, 2014).

**FIGURE 4:**
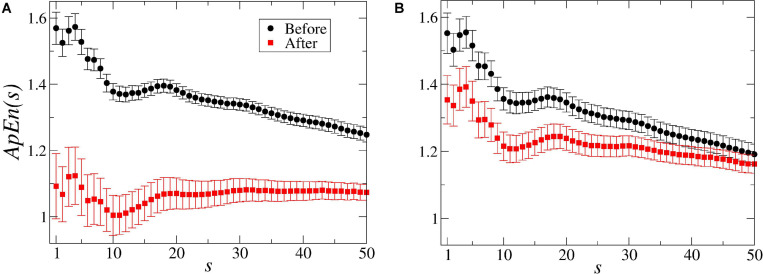
Multiscale entropy [*ApEn(s)*] values of the dodders’ electrome before and after being presented to their hosts. In **(A)**, for the bean treatment, the entropy [*ApEn(s = 1–50)*] reveals that the system was in a higher complexity state before the stimulus. After the presentation to the host, the system organises itself internally, reducing its complexity (in terms of randomness) by decreasing the entropy in all the scales. However, from scale 18 onward, there is a remarkable similarity with the pink (1/f) noise. In the dodders presented to the wheat plants **(B)**, the effect of the stimulus was less strong and, from scale 37 onwards, not significant. The bars represent standard error.

In average, the Approximate Entropy *ApEn*(*s* = 1) decreased from 1.56 ± 0.05 to 1.09 ± 0.10 after the stimulus in the bean treatment (see Figure 4A), and from 1.55 ± 0.06 to 1.35 ± 0.07 after the wheat treatment (Figure 4B), indicating a decrease in the complexity (irregularity) of the signals and an increase in the organisation (in terms of regularity). This decrease in the entropy values was observed for all the 50 scales of the *ApEn(s)* in the dodders presented to bean plants and only until scale 37 in the dodders presented to wheat plants (Figure 4).

The mean of all the parameters before and after the stimulus was compared between treatments (values for dodder + bean *before* were compared with dodder + wheat *before*, and values for dodder + bean *after* were compared with dodder + wheat *after*). As expected, there was no significant difference (*p* ≤ 0.05) in both treatments before the stimulus. However, a significant difference (*p* ≤ 0.05) was observed between the treatments in three parameters: autocorrelation, exponent β of the PSD, and the *ApEn(s)* (in the wheat treatment, only until scale 37).

#### Machine Learning Analyses

##### *Cuscuta* + bean before vs *Cuscuta* + bean after

**FIGURE 5:**
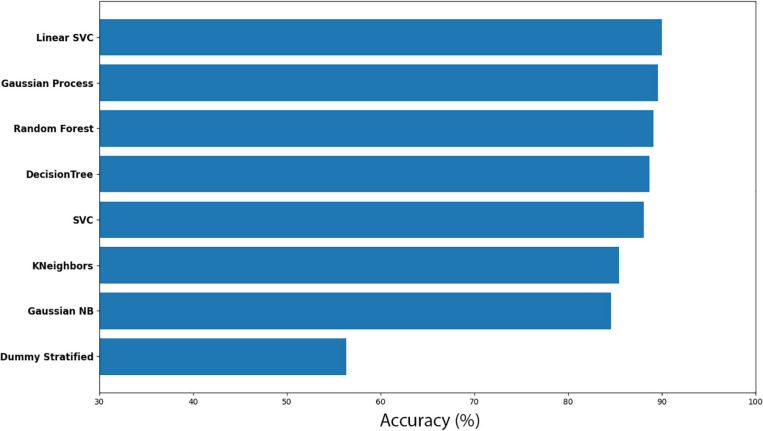
Accuracy for machine learning models for the dodders before and after being presented to the bean plant (CB-before vs CB-after).

**FIGURE 6:**
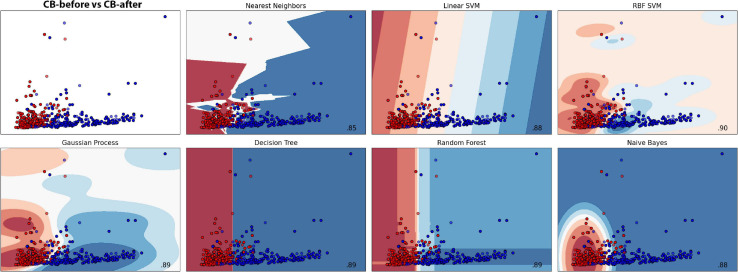
Machine learning classification results for each model in the dodder presented to beans. Red colour represents the dodders before being presented to the bean, and blue, after. Paler shades represent less accuracy for the classification.

Here, *Cuscuta* + bean before (CB-before) was compared with *Cuscuta* + bean after (CB-after). The accuracy measure for all the models employed are shown in Figure 5. An accuracy of 90.01% ± 5.44 was obtained with the model Linear SVC for separating these groups. When compared to the Dummy, which had an accuracy of 56.36% ± 13.16, it can be said that the result is excellent. Figure 6 shows the classification strategy used by each model. Four features of the total dataset were used for the training, and two were used as coordinates. There is a clear distribution of the groups, which contributed to the great performance of the machine learning (Figures 5, 6).

The results for all the models and their respective margin of error are presented in Supplementary Table 1. The models Gaussian Process and Random Forest obtained an accuracy very similar. However, we considered Random Forest as the second better model because of its smaller standard deviation. For the most efficient models, we analysed the Sensitivity and Precision using the True Positive and False Positive metrics for the choices of each group. This result is shown in Supplementary Table 2, which shows that Linear SVC obtained 86.95% against Random Forest’s 84.78% in Sensitivity. Nevertheless, Random Forest obtained a Precision rate higher than 92.85%, against 88.88% of Linear SVC’s. A perfect result would be 100% in both rates, which would indicate that the machine learning has learned to classify the samples perfectly. However, a perfect machine learning is hardly feasible. Therefore, it can be said that both models obtained an excellent success rate. With their accuracies, they learned to classify the groups CB-before and CB-after.

##### *Cuscuta* + wheat before vs *Cuscuta* + wheat after

**FIGURE 7:**
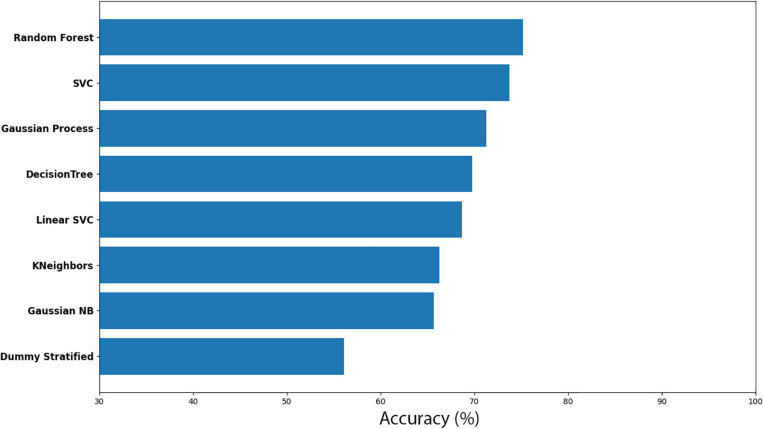
Accuracy for the machine learning models (CW-before vs CW-after).

**FIGURE 8:**
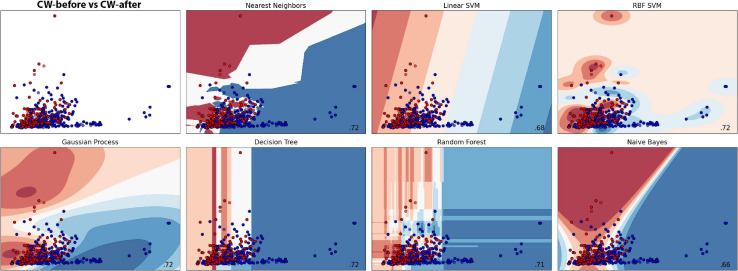
Machine learning classification results for each model in the dodder presented to wheat plants. Red colour represents the dodders before being presented to the wheat, and blue, after. Paler shades represent less accuracy for the classification.

Here, *Cuscuta* + wheat before (CW-before) was compared with *Cuscuta* + wheat after (CW-after). With the Random Forest method, we obtained an accuracy of 75.21% ± 7.41. The results for the accuracy models for all the models analysed are shown in Figure 7. Dummy obtained 56.08% ± 10.21 of accuracy. The results for all the models and their respective margin of error are presented in Supplementary Table 3. If we take into account the margin of error, Random Forest still is better than Dummy. The result of the machine learning was fairly good, considering that the model which does not learn had a worse result than the models which learn. The results of Sensitivity and Precision were quite expressive (Supplementary Table 4) and revealed that even with a smaller accuracy, both methods demonstrated that the features used were a good parameter for recognition. However, in one model there was a drop in Sensitivity, and in the other, in Precision, which resulted in smaller accuracy. The groups classification by the machine learning are shown in Figure 8.

##### *Cuscuta* + bean before vs *Cuscuta* + wheat before

The groups were divided into CB-before and CW-before. Supplementary Figures 4, 5 show how near the accuracy and data distribution is. Both groups cluster in the inferior region of the plot, which makes classification, in this case, extremely difficult. This is evidenced by the accuracy of only 60.45% ± 7.86 for the Random Forest model. There is no significant difference with Dummy’s accuracy of 56.08% ± 10.21. Sensitivity and Precision demonstrate that the results obtained by the best methods were bad. It evidences that the models were not able to classify the groups. Supplementary Table 5 shows the accuracy for each model and their margin of error, and Supplementary Table 6 shows Sensitivity and Precision of Random Forest and SVC.

##### *Cuscuta + bean* after *vs Cuscuta + wheat* after

**FIGURE 9:**
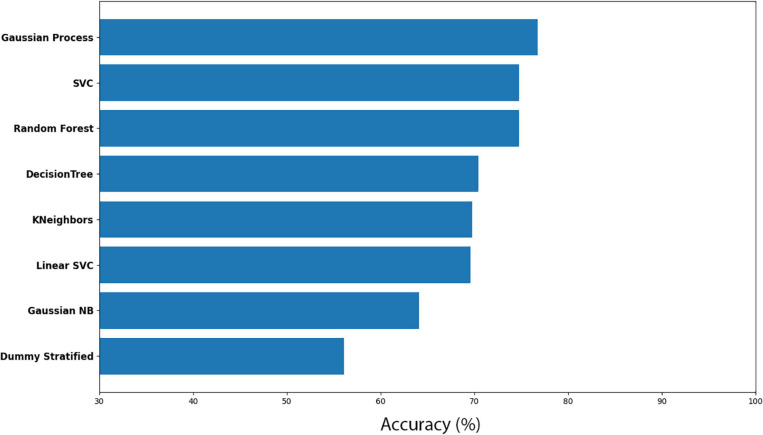
Accuracy for the machine learning models (CB-after vs CW-after).

**FIGURE 10:**
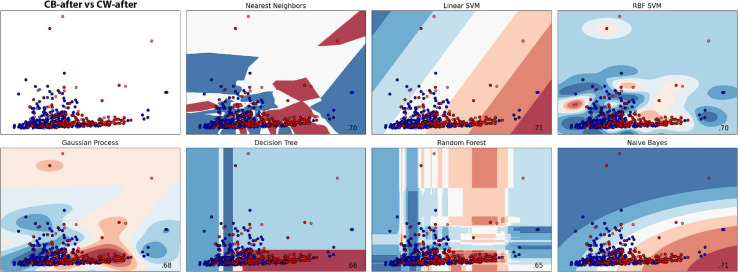
Machine learning classification results for each model in both treatments after the presentation to the hosts. Red colour represents the dodders after being presented to the bean, and blue, after being presented to wheat. Paler shades represent less accuracy for the classification.

The groups were divided into CB-after and CW-after and compared between them. The best model was the Gaussian Process, which obtained an accuracy of 76.73% ± 8.16, followed by SVC (74.79% ± 5.30) and Random Forest (74.70% ± 0.43). Dummy obtained only 56.08% ± 10.21 (Figures 9, 10 and Supplementary Tables 7, 8). In short, the machine learning was successful in distinguish group CB-after from CW-after.

##### Cuscuta *+ bean vs* Cuscuta *+ wheat*

Here we combined all the before and after samples for considering two groups into the wider categories: *Cuscuta* + bean (CB) and *Cuscuta* + wheat (CW). Random Forest model presented a low accuracy rate (margin of error ± 6.60%, see Supplementary Table 9). However, when compared to Dummy (accuracy of 53.80% ± 1.97), we can say that the machine learning had reasonable learning (Supplementary Figure 6).

Sensitivity and Precision (Supplementary Table 10) were not good, which demonstrates that the learning was from reasonable to bad within these groups.

##### Scatter plots

**FIGURE 11:**
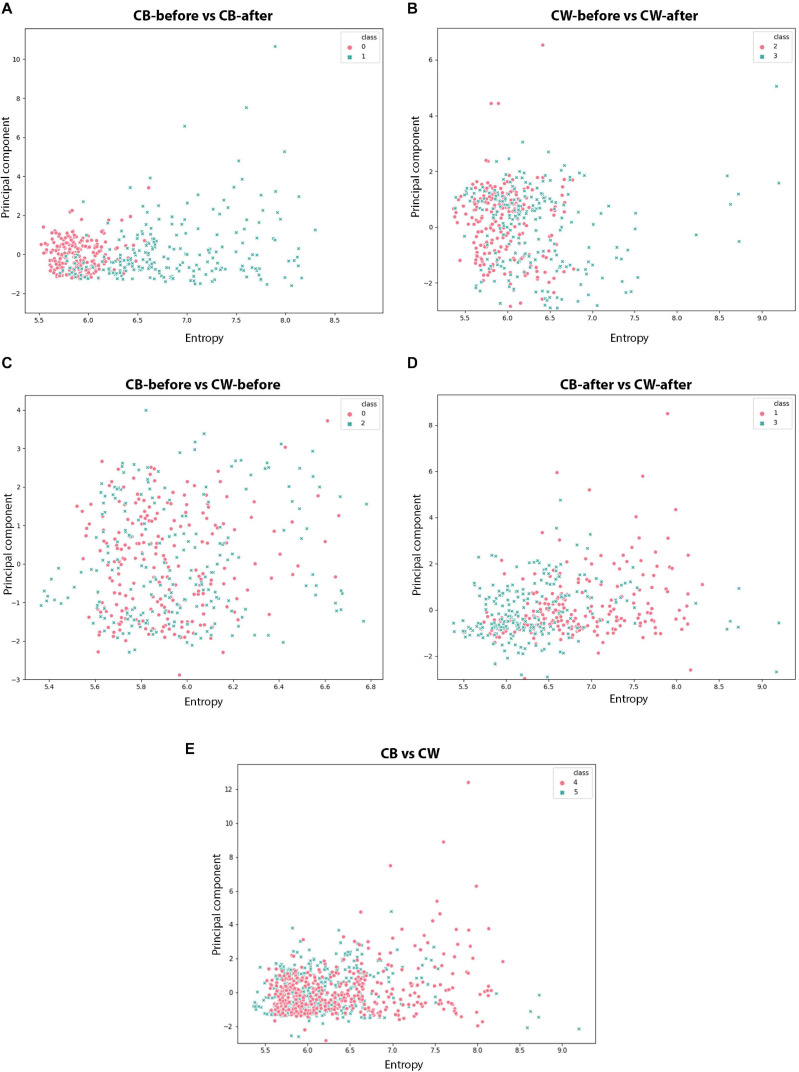
Scatter plots for the different time series analysed. **(A,B)**: red dots represent the values before the presentation to the host; blue dots represent the values after the presentation to the host. **(C–E)**: red dots represent dodders presented to beans, blue dots represent dodders presented to wheat plants.

**FIGURE 12:**
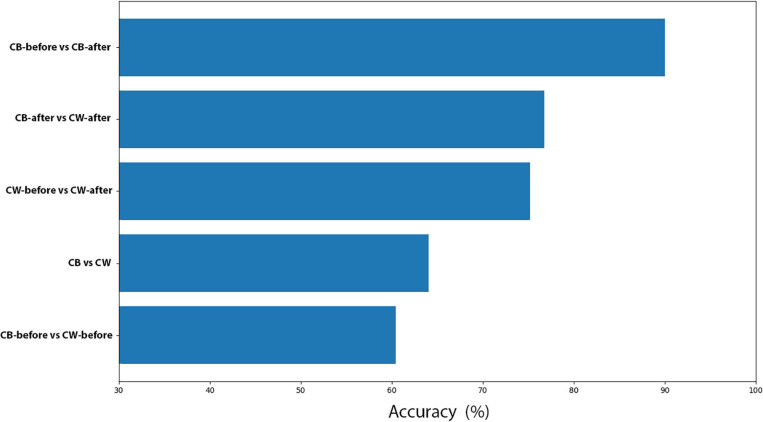
Accuracy for all the machine learning comparisons performed.

The scatter plots showed particular patterns for each stimulus. In CB-before and CB-after (Figure 11A), two groups can be neatly distinguished in the graph. For CW-before and CW-after (Figure 11B), this distinction is less clear, although existent. In CB-before and CW-after (Figure 11C), the points are utterly overlapped and scattered through the space, which immensely impairs its separation in groups. Finally, in CB-after and CW-after (Figure 11D), it is possible to see a separation of the dots in two different groups. Figure 11E shows the comparison between all the data for CB and CW. The accuracy of these measures is given in Figure 12, in which CB-before and CW-before is the group with the smaller accuracy, and CB-before and CB-after, the group with the highest accuracy.

## Discussion

The results of Experiment 1 showed that *C. racemosa* can perceive different hosts from a distance and changes its survival strategy depending on the specific host presented to it. The nutritional reserves of a *Cuscuta* twig detached from its host are limited and the plant cannot “afford” to waist them. Supposedly, if the dodder detects a host at a distance, there would be no need of synthesising so many pigments because it “foresees” there is a host ahead. On the other hand, if the dodder does not detect any host, it will need to prolong its survival, and activating photosynthetic pathways could be an alternative for it. Experiment 1 aimed to test this hypothesis.

In the total absence of hosts, the plants accumulated more photosynthetic pigments, which corroborates hypotheses 1 and 2. This increase in chlorophyll synthesis might be related to a need for photosynthesising more in order to avoid losing biomass through respiration. Our results add to the old and not solved discussion on whether photosynthesis has an important role to the life of *Cuscuta* (Peirce, 1894; MacLeod, 1961; Zimmermann, 1962; Pizzolongo, 1963; Pattee et al., 1965; Baccarini, 1966; Laudi, 1968; Laudi et al., 1974; Lyshede, 1985; Machado and Zetsche, 1990; Dinelli et al., 1993; Hibberd et al., 1998; Choudhury and Sahu, 1999; McNeal et al., 2007). Besides, we could say that the synthesis of different amounts of pigments depending on the host detected is a choice of the plant because it did it after processing information from the environment and not by an immediate mechanistic reaction. It changed its behaviour in a goal-oriented, non-automatic manner. Presumably, as a strategy for increasing its probability of survival (Trewavas, 2016; Calvo et al., 2020). Recent studies with climbing plants have been confirming that plants are deeply aware of their environment. Plants act with the available environmental cues and make choices and behave in goal-oriented manners to increase their chances of survival (Gianoli and Carrasco-Urra, 2014; Guerra et al., 2019; Ceccarini et al., 2020).

Unexpectedly, the treatment which led to the smallest content of chlorophylls was the wheat treatment. It also resulted in the smallest shoot length. It makes us wonder whether, in this case, the wheat is inhibiting the synthesis of photosynthetic pigments in some unknown interaction from a distance, perhaps for protection, although we cannot speculate more without further tests. We also were surprised by the fact that the dodders presented to the sticks and the beans accumulated similar amounts of pigments. Anyhow, the dodder plants used in this experiment seem to be able to detect different kinds of hosts and respond according to each kind with physiological adjustments. It, at least partially, supports our third hypothesis.

With the electrophysiological results, we corroborated hypotheses 4 and 5. The dynamic in the dodders’ electrome before and after they were presented to different hosts changed consistently, and the changes were distinguished for each kind of host. Besides, it seems that the undulating pattern of the time series is a characteristic response of the dodder plant used in this experiment when presented to their hosts, especially a viable one.

The changes in the electrome dynamics were enabled by the presence of the host, and not by other factors such as manipulation of the boxes or the change of air when the boxes were opened. The new dynamic (undulating) appeared minutes after introducing the host in the box (about 15–20 min later). Otherwise, the changes in the electrome would be immediate. If the VOCs of the host were causing this change in the electrome, maybe a certain amount of time is required for they accumulate in concentration enough to cause the observed changes.

The drastic increase in the average correlation time *L* in both treatments (but, especially, in the dodders presented to the viable host) was accompanied by a general decrease in the *ApEn(s)* values and the value of the β exponent of the PSD (not significant for the viable host). This means that the signals became more regular and organised, which can explain the increase in the autocorrelation. It suggests a higher “coordination” of the electrical signals (Saraiva et al., 2017).

Normally, decreases in the complexity of the signals—verified, e.g., by an increase in the β exponent of PSD—suggest sick plants or plants under strong stress. Souza et al. (2017) showed that in soybean plants, low light, cold, and hydric stress by mannitol caused an increase of the β exponent from approximately 1.51 ± 0.21 (a reddened noise) to 1.96 ± 0.30 in plants under low light, 2.85 ± 0.69 in plants under cold stress, and 2.58 ± 0.34 in plants under osmotic stress (Souza et al., 2017). The first case describes a noise next to brown (β = 2) and the second and third, a noise next to black (β = 3). In another study, Saraiva et al. (2017) observed that soybean plants had an exponent β of 1.5 ± 0.3 before, and 2.6 ± 0.2 after being stressed with mannitol. Consonant with these studies, dodders presented to wheat showed the major decrease in the β exponent, which could suggest that they were under tougher stress than those presented to the bean plants.

On the other hand, when the stimulus is subtle and non-destructive, the complexity of the signals seems to increase. Simmi et al. (2020) studied tomato plants infected by the powdery mildew (*Oidium* sp.), a biotrophic fungus. The value of the β exponent before the inoculation of the fungus was 2.13, which indicates a brown noise, and decreased to β = 1.95 after the inoculation, presenting higher complexity and more information organised in higher temporal scales.

In the present study, interestingly, the mean value of the β exponent for the signals recorded from the dodders was fairly close to the 1/*f* noise (pink noise), especially before the stimulus. As a matter of fact, the β exponent before the stimulus was the closest from the pink noise than in any of the studies cited before. This kind of noise is the most related to highly organised, complex, scale-invariant and critically self-organised processes (He et al., 2010; He, 2014). It must be noted, however, that this does not represent the typical “basal” noise of the dodders, but rather the noise of plants detached from their hosts and severely pruned. That is, under strong stress. The *ApEn(s)* also corroborates this finding. A decrease in the complexity of the electrome in the first 10 scales before the presentation to the hosts indicates that, in terms of organisation and complexity, the system is already compromised in lower scales.

The results with the *ApEn* and the *ApEn(s)* demonstrate that there is a decrease in the electrome’s complexity after the presentation to the hosts. Before this stimulus, both curves had the same shape, slope and values, indicating that the electromes’ dynamics were equivalent between treatments. However, when the dodders were presented to the beans, the decrease in the *ApEn* values was significant in all the scales. For the dodders presented to the wheat, it was only significant until scale 37 (Figure 4). It demonstrates that, especially in the bean treatment, the presence of the host is affecting the dodders’ electrome dynamics in all the scales. The subtle slope depicted in the graphs also evidences a tendency toward the 1/*f* noise (pink noise), especially in higher scales, which corroborate the results obtained by the calculation of the β exponent of the PSD. Interestingly, in the bean treatment, the shape of the graph from scale 18 to upper scales indicates an undeniable similarity with 1/*f* noise (Costa et al., 2005).

The decrease of complexity verified by the decrease of the exponent β and the *ApEn(s)*, accompanied by an increase in the correlation time *L* suggests that there was a higher regularity in the voltage variation runs. It makes us hypothesise that, after being exposed to a host in potential, dodders coordinate their bioelectrical activity, focusing on the stimuli coming from a likely source of resources. It can be suggested that the plant is “paying attention” to an informative environmental cue that did not exist before and, suddenly, came to exist. Furthermore, the dodder is coordinating a response to this specific cue (which is suggested by the higher values of the average correlation time *L*). We know that, in the long term, the dodder will change its behaviour depending on the kind of host perceived, not only thanks to the studies of pioneering researchers (Kelly, 1990, 1992; Koch et al., 2004; Benvenuti et al., 2005; Runyon et al., 2006) but also by the results obtained with Experiment 1 in the present work.

By “attention” in plants, we mean what was proposed by Marder (2012, 2013). It is a “disproportionate investment of physical or mental energy by an organism, tissue, or cell into a particular activity or into the *reception of a singled-out stimulus or set of stimuli*.” (Marder, 2013, our emphasis). Our results on the electromic responses of *C. racemosa* when looking for a host, as far as we know, could be the first empirical evidence for a process of attention in plants, involving higher regularity (focusing) on plant bioelectrical activity.

The investment of physical energy by the dodders manifested in the highest ΔV and in the increase of the average correlation time *L* of the ΔV events, the higher regularity of them, and in its proximity to noises of high complexity and levels of information suggest that dodders indeed perceive a potential host and change their bioelectrical behaviour accordingly. Moreover, the significant differences (*p* ≤ 0.05) in correlation time *L*, *ApEn(s)* and the exponent values β of the PSD suggest that, in fact, the dodder recognises different kinds of hosts and reacts to them differently, already in the electrophysiological level. It was also corroborated by the results of the machine learning analyses, which will be discussed ahead.

More recently, Grondin (2016) defined attention in a similar, although less precise, way: attention is “the process allowing to become aware of a few things and to capture a part, admittedly very limited, of what is going on around.” (Grondin, 2016). Plants are aware of their environment (Trewavas, 2016; Guerra et al., 2019; Calvo et al., 2020; Ceccarini et al., 2020) and they must be if they are to survive in an ever-changing environment where different parameters fluctuate over time. Attention is especially necessary for unattached parasitic plants because their nutrition normally comes from one single source. For autotrophic plants, the process of attention might be more diffused and difficult to observe empirically because each module is focusing on the most important cue or signal for them at the moment (Trewavas, 2003; Lüttge, 2019). So, dodder plants, and other parasitic plants, can be a good model for studying the phenomenon of attention in plants.

The results obtained with the analyses by machine learning of the time series also corroborated the hypotheses 4 and 5. They clearly demonstrate that the electrical signalling of the dodders (which was indistinguishable between the treatments before the stimuli) changed considerably when the plants detected a host nearby. As for the electrophysiological analyses, the response of the dodders presented to the beans—the viable hosts—were much more intense and precise, thus easily recognisable for the machine learning with the highest accuracy. The pattern of the time series, or “electrical signature” (Simmi et al., 2020) of the dodders was clearly different depending on the species of the host presented. It demonstrates that the dodder recognises different species of hosts from a distance. The accuracy in separating the groups CB-after from CW-after was even higher than the accuracy for the separation of the groups CW-before from CW-after. In this case, the electrical activity of the dodder is not so different when presented to the wheat. This milder activity was also detected by the electrophysiological analyses. However, it was enough for the machine learning to learn how to differentiate both groups.

Machine learning techniques have been increasingly used for studying plant physiology and behaviour (e.g., Ma et al., 2014; Shaik and Ramakrishna, 2014; Behmann et al., 2015; Singh et al., 2016). It is also an excellent technique for helping to understand the meaning of the diverse patterns of the electrome that emerge when the plant is interacting with different aspects of its environment (Pereira et al., 2018; Simmi et al., 2020). This study supports the machine learning as a valid tool for studying the ecology and physiology of plants and demonstrates that even interactions from a distance are enough for causing drastic effects in the electrical signalling dynamics.

The results of this study also corroborate the claim that the interface between the internal and external environment of the dodders is, indeed, an electrical interface as suggested by Debono and Souza (2019). After all, most of what a plant perceives is, somehow, mediated by some kind of electrical signalling (Fromm and Lautner, 2007; Huber and Bauerle, 2016; Canales et al., 2018; Debono and Souza, 2019). Furthermore, our results suggest that the “preuptake mechanism” proposed by Koch et al. (2004), and the process of choosing in *Cuscuta*, have a bioelectrical basis, for the earlier sign of host detection, before any other phenotypic and/or behavioural change, were the alterations in the electrome.

It was not the aim of this work to individualise the means by which dodders perceive the hosts. However, according to the literature available, it seems plausible to us that the main way by which the dodders perceived the hosts was by VOCs (Runyon et al., 2006). It led us to speculate how the VOCs mediating the interaction of the plants would cause the observed alterations in the electrome.

It is known that *P. vulgaris* emits constitutively at least two different VOCs: (3*E*)-4,8-dimethyl-1,3,7-nonatriene (DMNT) and (Z)-3-hexenyl acetate (Wei et al., 2006; Sufang et al., 2013). In a study with tomato plants (*Solanum lycopersicum* L. cv. Micro-Tom)—a plant from the same order of the dodders (Solanales)—exposure to (Z)-3-hexenyl acetate caused depolarisation of the mesophyll’s cell membranes and led to the accumulation of cytosolic Ca^2+^ (Zebelo et al., 2012). Additionally, a study with Arabidopsis [*Arabidopsis thaliana* (L.) Heynh.] demonstrated that the perception of the VOC (*E*)-4,8-dimethyl-1,3,7-nonatriene [(*E*)-DMNT] caused an accumulation of apoplastic Ca^2+^ in the cytosol (Asai et al., 2009). In both studies, the responses to VOCs are directly related to the mechanisms of generation and propagation of electrical signals in plants (de Toledo et al., 2019). Hence, it could be hypothesised that there are membrane receptors for these and, maybe, other VOCs which trigger electrophysiological responses, such as those reported here. It could be the first step in an explanation for how VOCs alter the internal electrical signalling of dodders. This opens a worthwhile avenue of future investigation.

Furthermore, other means of perception are worthy of being explored. Light cues are recognised as important for dodder foraging (Orr et al., 1996; Benvenuti et al., 2005; Wu et al., 2019). Additionally, in recent years, Haberlandt’s plant ocelli hypothesis has been revisited with new pieces of evidence (Haberlandt, 1905; Baluška and Mancuso, 2016; Mancuso and Baluška, 2017). Parasitic plants rely on its senses for detecting hosts, and perhaps vision or some analogue sense could be of use. By combining the methodology used in this work with experiments devoted to testing the plant ocelli hypothesis, dodder plants may stand as a good plant model for advancing in this new research field.

Concluding, the interaction from a distance with other plants caused changes in the physiology of the parasitic plant *C. racemosa*. When they detected a host, the dodders refrained themselves of synthesising chlorophylls, thus changing its survival strategy. Besides, it challenges the idea that the low content of chlorophylls in the dodders is due to photodestruction, which was proposed years ago (Choudhury and Sahu, 1999; Sahu and Choudhury, 2000). This work also found empirical evidence for a process of attention in a plant, which deserves to be better studied in the future. Electrophysiological analyses seem promising as a tool for investigating this phenomenon in plants. Finally, perhaps more complex processes, mediated by electrical signals among others, are taking place in the plant, driving foraging strategies. The path to be followed invites us to seek an understanding of how these two levels, the bioelectrical and behavioural ones, are connected between them. There is still so much to learn from the behaviour of these fascinating plants.

## Data Availability Statement

The original contributions presented in the study are included in the article/Supplementary Material, further inquiries can be directed to the corresponding author/s.

## Author Contributions

AP designed the experiments and wrote the manuscript. AP, GR, and LB conducted all the experiments. All the authors analysed, interpreted, and discussed the data. TO did the machine learning analyses and wrote most of the parts concerning it. GS supervised the work and contributed to the manuscript writing. All the authors reviewed the manuscript and contributed to it.

## Conflict of Interest

The authors declare that the research was conducted in the absence of any commercial or financial relationships that could be construed as a potential conflict of interest.
