# Supplementary material for: Detection of Different Hosts From a Distance Alters the Behaviour and Bioelectrical Activity of Cuscuta racemosa
Source: Front Plant Sci. 2021 Mar 18;12:594195. doi: 10.3389/fpls.2021.594195 (PMC8012508; doi:10.3389/fpls.2021.594195)
Supplement: Supplementary file 1 [file Data_Sheet_1.docx]

Supplementary Material

**Mathematical description of the electrophysiological analyses**

After visual checking of the time series, they were analysed by Fast Fourier Transform (FFT) for observing the dominant frequencies of the ΔV events (Saraiva et al., 2017; Souza et al., 2017), and by the Wavelet Transform $\mathbb{W}$, which shows the amplitudes $\mathbb{W(}\Delta V)$ versus frequencies *f* and time *t*. The wavelet transform is already used for analysing neurophysiological time series in humans and non-human animals (Adeli et al., 2003; Hramov et al., 2015). Thus, a histogram for each time series was made for verify the shape of probability distribution function (PDF) of $\mathbb{\Delta V}$. For a better understanding of the distribution of events in the histograms, the skewness and kurtosis of the time series was determined.

Consider *x* as a set of random variables $x=\{x_{i}, i=1,\ldots,N\}$. For gaussian distribution, the skewness ($\alpha$) and kurtoses (κ) are 0,0 (symmetric distribution) and 3,0, respectively. Its definition is: $\alpha=<{(x-\lambda)}^{3}>/\sigma^{3}$ and $\kappa=<{(x-\lambda)}^{4}>/\sigma^{4}$, where $\lambda=<x>=1/N\cdot\sum_{i=1}^{N} x_{i}$ mean value, $\sigma=\sqrt{{<(x-\lambda)}^{2}>}$ standard deviation. The autocorrelation function (Pearson correlation) was analysed for verifying the correlation of a point in the series with its previous points, following the equation: $\rho\left( \tau\right)= <\left( x_{\tau}-<x> \right)\cdot\left( x-<x> \right)>/\left( \sigma_{\tau}\cdot\sigma\right)$ , $-1\leq\rho\leq1$ and $\tau$ is the time lag. When $\rho\left( \tau\right)=0$, there is no linear correlation between $x(t)$ and $x_{\tau}(t-\tau)$. When $\rho\left( \tau\right)>0$, events in the past $x(t)$ positively influence the future $x_{\tau}(t-\tau)$, and when $\rho\left( \tau\right)>0$, the opposite occurs (Simmi et al., 2020). The average correlation time is defined by $L=\int\tau\cdot\left| \rho\left( \tau\right) \right|d\tau/\int\left| \rho\left( \tau\right) \right|d\tau$. We also calculated the Probability Density Function (PDF), as mentioned before. We have found that the function which describes the behaviour of $|\Delta V|$ in plant electrophysiological time series is a power law, which is associated with scale invariance for amplitude $|\Delta V|$, ${f(|\Delta V|)\sim|\Delta V|}^{-\mu}$, $1<\mu<3$ (Saraiva et al., 2017; Souza et al., 2017; Simmi et al., 2020).

The Power Spectral Density (PSD) was calculated for understanding how the power (energy/intensity) is distributed through the frequencies *f*. This PSD decay follow a power law $PSD\sim1/f^{\beta}$ on the asymptotic limit $(f\to\infty)$. We calculate the exponent *β*  by a linear regression, taking the square of the fast Fourier transform (FFT). The PSD is obtained mathematically by the Fourier transform of autocorrelation function by Wiener–Khinchin theorem $PSD\left( \omega\right)=\int_{-\infty}^{+\infty} \rho\left( \tau\right)e^{-i\omega\tau}d\tau$ , with $\omega=2\pi f.$

The complexity of the electrome was estimated by the approximate entropy (*ApEn*) (Pincus, 1991, 1995) and the multiscale sample entropy [*ApEn(s)*] (Costa et al., 2005). The *ApEn(s),* with the scale *s* assuming integer values ​​*s = 1, 2, 3*, ..., consists of dividing the time series $\mathbb{\Delta V=\{}{\Delta V}_{1}, {\Delta V}_{2}, \ldots, {\Delta V}_{N}\}$ into blocks of size *s*. For *s = 1*, we have *ApEn = ApEn(s=1*). See an example of how the method works: for *s = 2*, the new times series become $\mathbb{\Delta V}\left( s=2 \right)=\{\left( {\Delta V}_{1}+{\Delta V}_{2} \right)/2,\left( {\Delta V}_{3}+{\Delta V}_{4} \right)/2,\ldots,(\left( {\Delta V}_{N-1}+{\Delta V}_{N} \right)/2\}$, with $N/2$ elements, and so on for *s = 3, 4,....*The next step is building the *M* vectors in the embedding dimension *m > 0* and time lag *τ ≥ 0,* both integer values*,* (Takens, 1981) ${\Delta V}_{j}={(\Delta V}_{j},{\Delta V}_{j-\tau},{\Delta V}_{j-2\tau},...,{\Delta V}_{j-(m-1)\tau})$ with *j=1,2,...,M*, such that $M=N-(m-1)\tau$. In **Supplementary Figure 1A** (white noise) and **Supplementary Figure 1C** (correlated noise), two examples are shown for *m = 2 dimension (x versus y)*, where each dot on the graph represents a vector. For each vector ${\Delta V}_{j}$ the number of neighbours ${N(j)}_{neig\leq r}$ is calculated within a hypersphere of radius *r*. After that, we take the average of the logarithm of the number of neighbours, which is given by $\phi^{m}\left( r,s \right)=\frac{1}{M}\sum_{j=1}^{M} ln({N(j)}_{neig\leq r})$. Finally, the multiscale sample entropy is

$ApEn(m,r,s)=\phi^{m}\left( r,s \right)-\phi^{m+1}\left( r,s \right)$.

In this work we fix *m=2*, $\tau=1$ and $r=0.2\cdot\sigma$ (Pincus, 1991), where $\sigma$ is the standard deviation from $\mathbb{\Delta V=\{}{\Delta V}_{1}, {\Delta V}_{2}, \ldots, {\Delta V}_{N}\}$.

**References**

Adeli, H., Zhou, Z., Dadmehr, N. (2003) Analysis of EEG records in an epileptic patient using wavelet transform. J. Neurosci. Methods. 123: 69–87. doi: 10.1016/S0165-0270(02)00340-0

Costa, M., Goldberger, A.L., Peng, C.-K. (2005). Multiscale entropy analysis of biological signals. Phys. Rev. 71, 1–18. doi: 0.1103/PhysRevE.71.021906

Hramov, A.E., Koronovskii, A.A., Makarov, V.A., Pavlov, A.N., Sitnikova, E. (2015). Wavelets in neuroscience. Heidelberg, Germany: Springer-Verlag Berlin Heidelberg.

Saraiva, G.F.R., Ferreira, A.S., Souza, G.M. (2017). Osmotic stress decreases complexity underlying the electrophysiological dynamic in soybean. Plant Biol. 19, 702–708. doi: 10.1111/plb.12576

Souza, G.M., Ferreira, A.S., Saraiva, G.F.R., de Toledo, G.R.A. (2017). Plant “electrome” can be pushed toward a self-organized critical state by external cues: evidences from a study with soybean seedlings subject to different environmental conditions. Plant Signal. Behav. 12:e1290040. doi: 10.1080/15592324.2017.1290040

Simmi, F.Z., Dallagnol, L.J., Ferreira, A.S., Pereira, D.R., Souza, G.M. (2020). Electrome alterations in a plant-pathogen system: toward early diagnosis. Bioelectrochemistry 133:107493. doi: 10.1016/j.bioelechem.2020.107493

Pincus, S. (1995). Approximate entropy (ApEn) as a complexity measure. Chaos 5, 110–117. doi: 10.1063/1.166092

Pincus, S.M. (1991). Approximate entropy as a measure of system complexity. Proc. Natl. Acad. Sci. USA. 88, 2297–2301. doi: 10.1073/pnas.88.6.2297

Takens, F. (1981). “Detecting strange attractors in turbulence,” in Dynamical Systems and Turbulence, eds A. Rand and L. S. Young (Berlin: Springer), 366–381. doi: 10.1007/bfb0091924


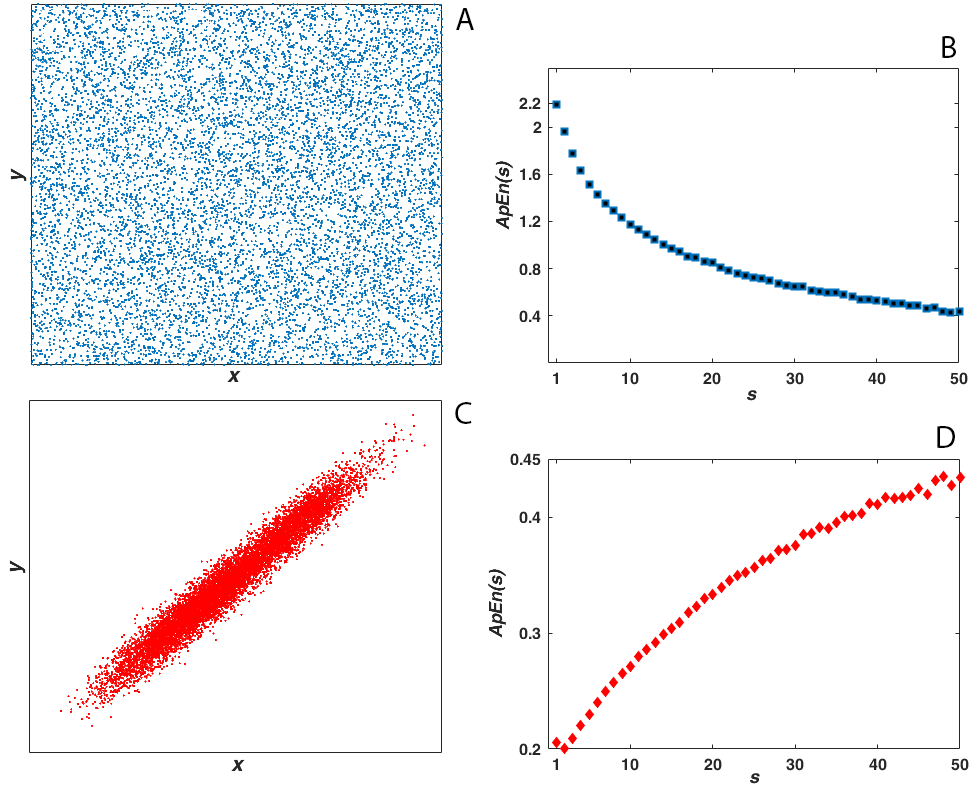


**Supplementary Figure 1.** In **(A)** and **(C)**, each point on the graph represents a vector in two dimensions. For white noise **(A)**, the vectors are uniformly distributed in the phase space. In this case, entropy *ApEn(s)* decreases with the *s* scale **(B)**. For correlated processes **(C)**, the array of vectors reveals the degree of linear correlation between events x versus y. In this situation, entropy *ApEn(s)* **(D)** always increases with the *s* scale.

**
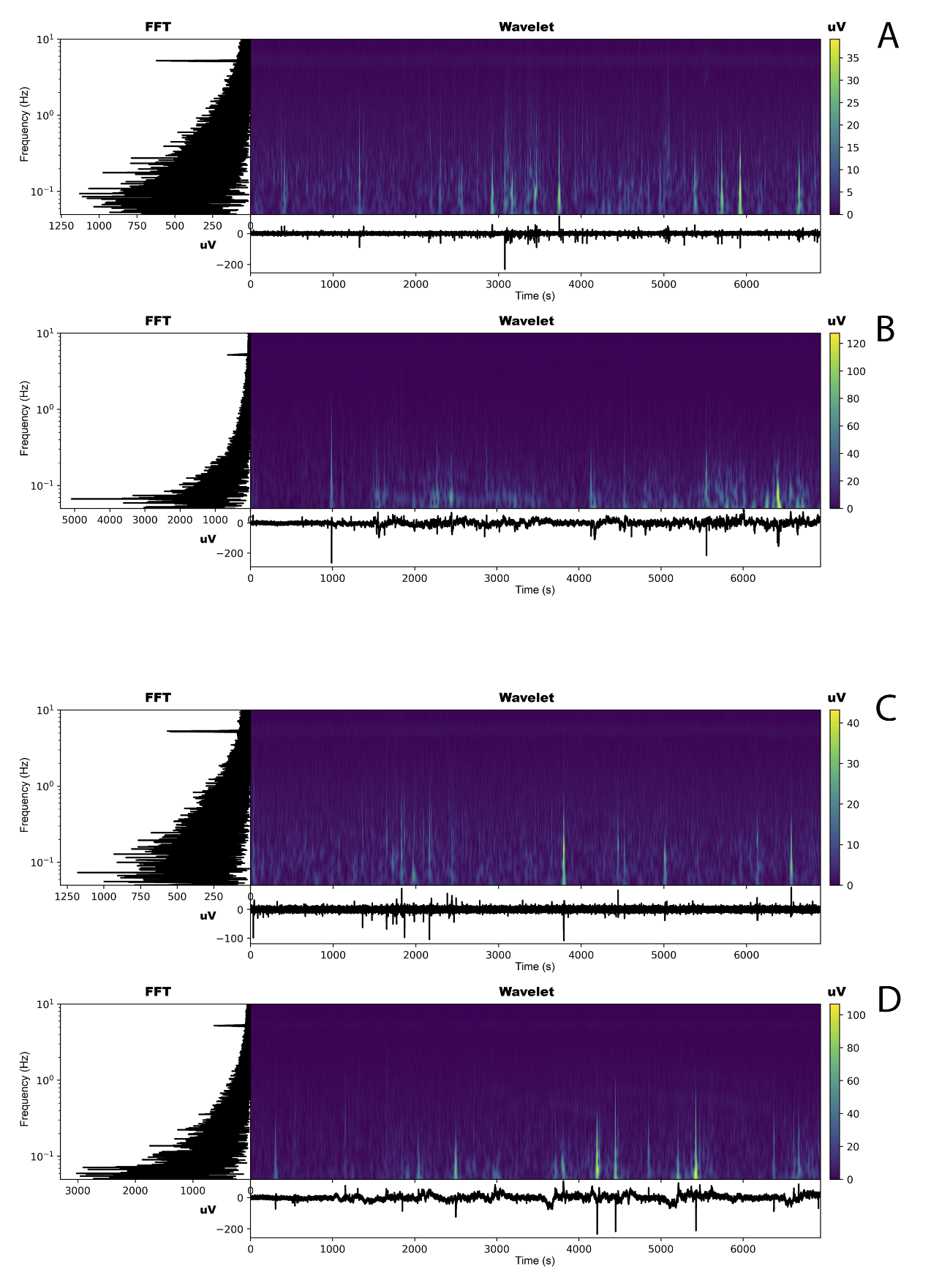
**

**Supplementary Figure 2.** Time series, fast Fourier transform in log-linear scale, and wavelet transform for the dodder’s electrome before (**A** and **C**) and after **(B** and **D**) being presented to bean.

**
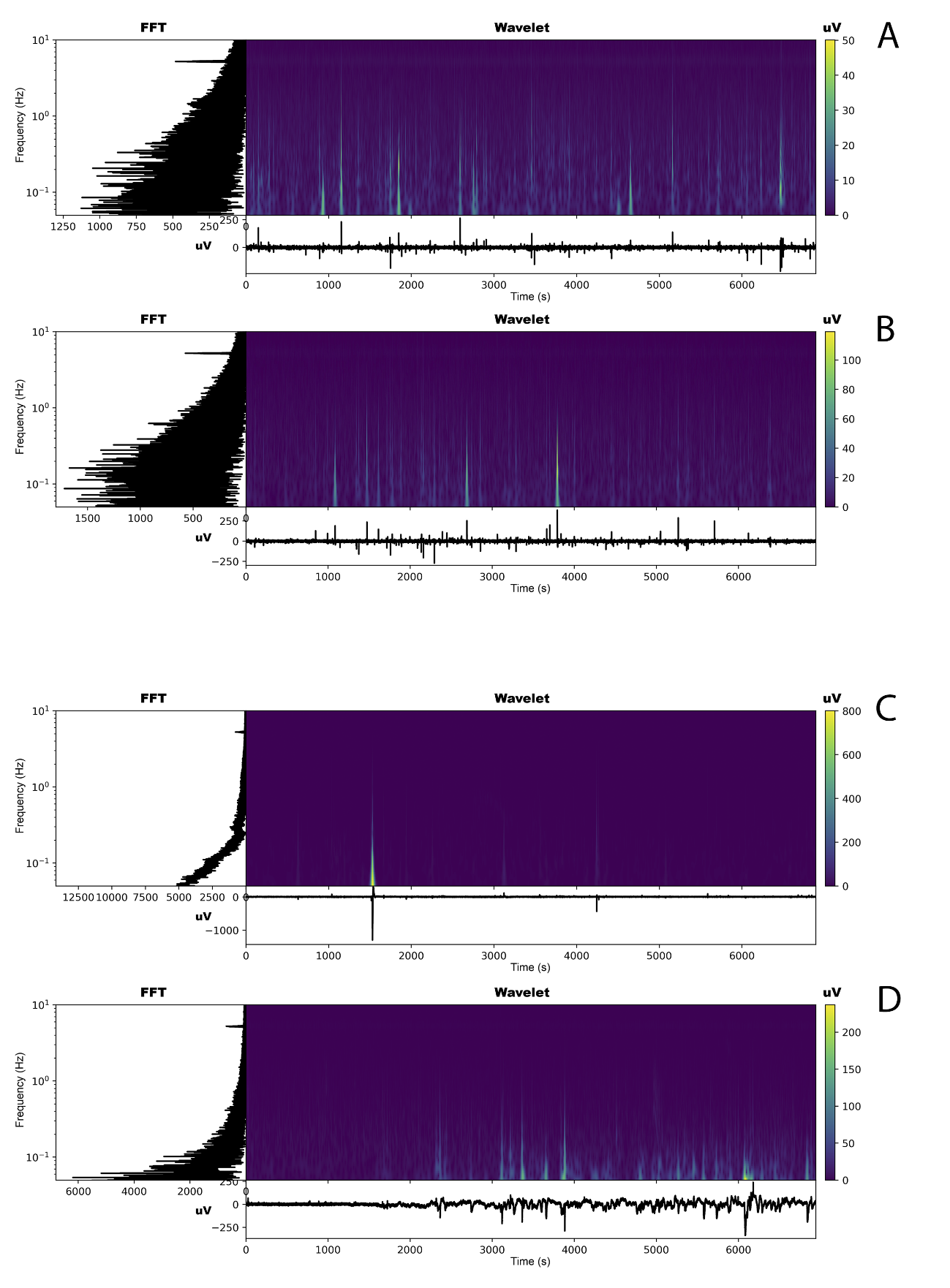
**

**Supplementary Figure 3.** Time series, fast Fourier transform in log-linear scale, and wavelet transform for the dodder’s electrome before (**A** and **C**) and after (**B** and **D**) being presented to a wheat plant.

**Supplementary Table 1.** Results for the accuracy of all the machine learning models with standard deviation (STD) for the classification of the time series in the groups before and after the dodder’s presentation to the bean plants.

| **Models** | **Accuracy (%)** | **STD (%)** |
| --- | --- | --- |
| Linear SVC | 90.01 | ± 5.44 |
| Gaussian Process | 89.56 | ± 5.56 |
| Random Forest | 89.13 | ± 0.86 |
| DecisionTree | 88.71 | ± 3.50 |
| SVC | 88.05 | ± 6.10 |
| KNeighbors | 85.43 | ± 2.77 |
| Gaussian NB | 84.56 | ± 2.34 |
| Dummy Stratified | 56.36 | ± 13.16 |

**Supplementary Table 2.** Comparison of the two best machine learning models for the classification of the time series in the groups before and after presentation to the bean plants.

| **Parameter** | **Linear SVC** | **Random Forest** |
| --- | --- | --- |
| Error (%) | 11.95 | 10.86 |
| Sensitivity (%) | 86.95 | 84.78 |
| Precision (%) | 88.88 | 92.85 |

**Supplementary Table 3.** Results for the accuracy of all the machine learning models with standard deviation (STD) for the classification of the time series in the groups before and after the dodder’s presentation to the wheat plants.

| **Models** | **Accuracy (%)** | **STD (%)** |
| --- | --- | --- |
| Random Forest | 75.21 | ± 7.47 |
| SVC | 73.75 | ± 8.56 |
| Gaussian Process | 71.28 | ± 3.64 |
| DecisionTree | 69.78 | ± 2.90 |
| Linear SVC | 68.69 | ± 10.34 |
| KNeighbors | 66.30 | ± 3.60 |
| Gaussian NB | 65.65 | ± 7.32 |
| Dummy Stratified | 56.08 | ± 10.21 |

**Supplementary Table 4.** Comparison of the two best machine learning models for the classification of the time series in the groups before and after presentation to the wheat plants.

| **Parameter** | **Random Forest** | **SVC** |
| --- | --- | --- |
| Error (%) | 22.82 | 21.73 |
| Sensitivity (%) | 80.43 | 69.56 |
| Precision (%) | 75.51 | 84.21 |

**
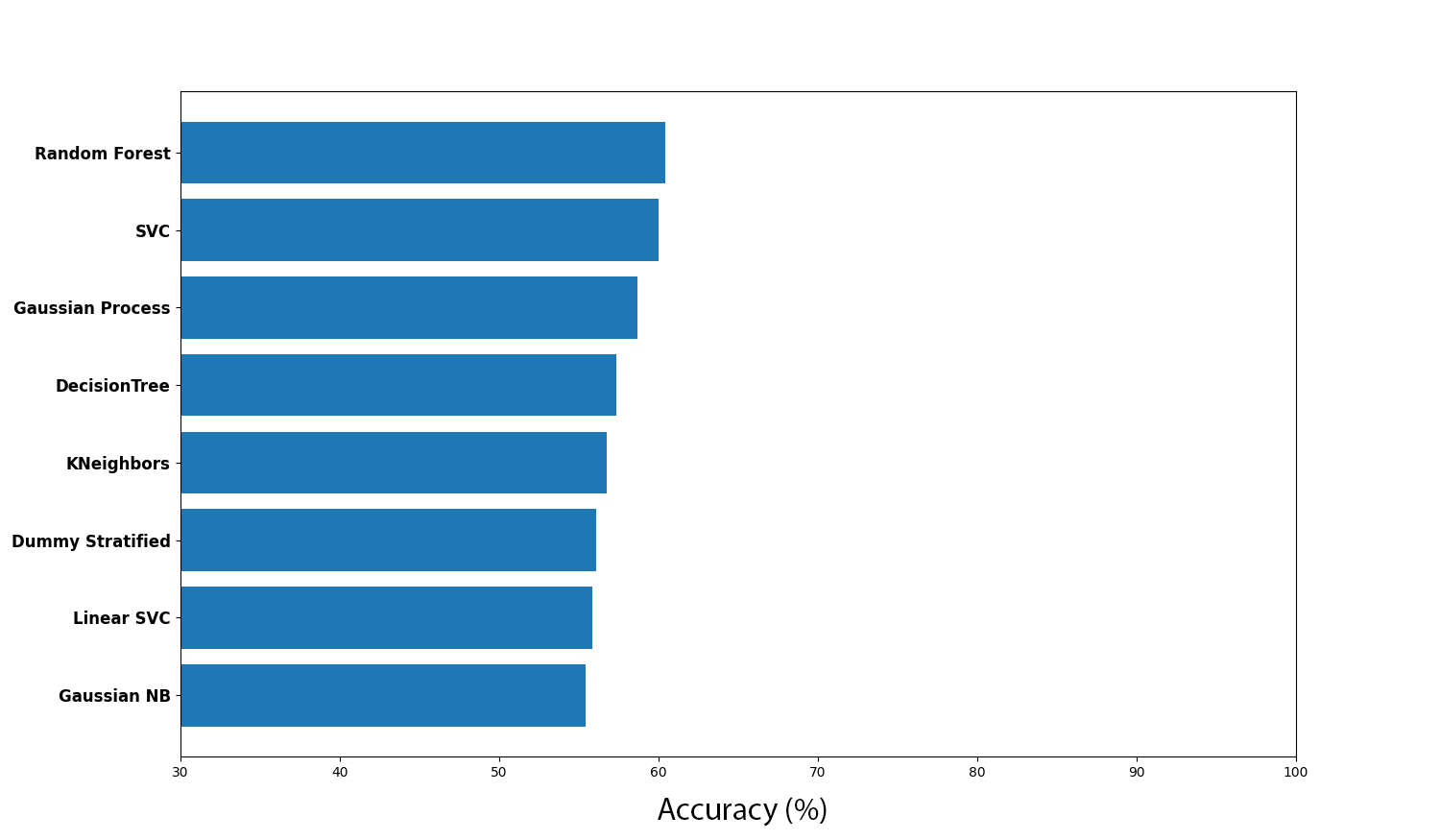
**

**Supplementary Figure 4.** Accuracy for machine learning models for the dodders before being presented to the hosts (CB-before vs CW-before).

**
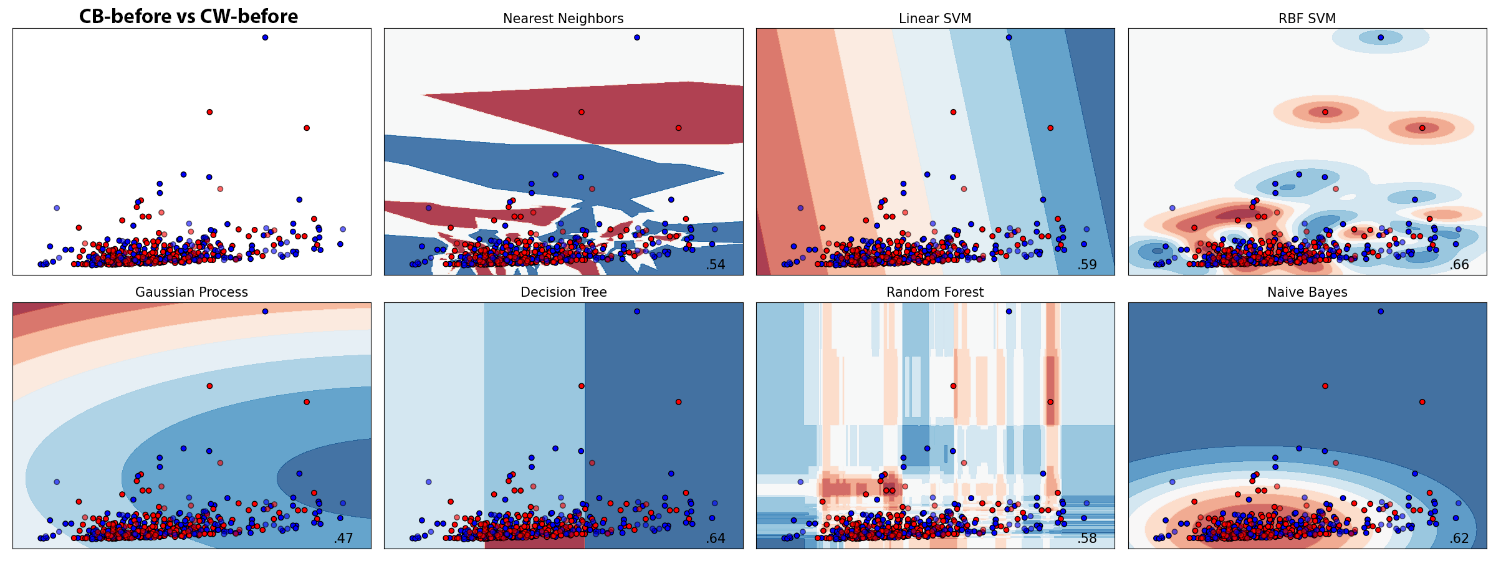
**

**Supplementary Figure 5.** Machine learning classification results for each model in both treatments before the presentation to the hosts. Red colour represents the dodders before being presented to the bean plants, and blue, before being presented to the wheat plants. Paler shades represent less accuracy for the classification.

**Supplementary Table 5.** Results for the accuracy of all the machine learning models with standard deviation (STD) for the classification of the time series in the groups CB-before and CW-before.

| **Models** | **Accuracy (%)** | **STD (%)** |
| --- | --- | --- |
| Random Forest | 60.45 | ± 7.86 |
| SVC | 60.00 | ± 4.83 |
| Gaussian Process | 58.69 | ± 8.74 |
| DecisionTree | 57.39 | ± 2.81 |
| KNeighbors | 56.73 | ± 3.39 |
| Dummy Stratified | 56.08 | ± 10.21 |
| Linear SVC | 55.88 | ± 6.60 |
| Gaussian NB | 55.43 | ± 3.76 |

**Supplementary Table 6.** Comparison of the two best machine learning models for the classification of the time series in the groups CB-before and CW-before.

| **Parameter** | **Random Forest** | **SVC** |
| --- | --- | --- |
| Error (%) | 39.13 | 38.04 |
| Sensitivity (%) | 58.69 | 39.13 |
| Precision (%) | 61.36 | 72.00 |

**Supplementary Table 7.** Results for the accuracy of all the machine learning models with standard deviation (STD) for the classification of the time series in the groups CB-after and CW-after.

| **Models** | **Accuracy (%)** | **STD (%)** |
| --- | --- | --- |
| Gaussian Process | 76.73 | ± 8.16 |
| SVC | 74.79 | ± 5.30 |
| Random Forest | 74.70 | ± 0.43 |
| DecisionTree | 70.43 | ± 1.98 |
| KNeighbors | 69.78 | ± 1.46 |
| Linear SVC | 69.56 | ± 0.86 |
| Gaussian NB | 64.11 | ± 6.63 |
| Dummy Stratified | 56.08 | ± 10.21 |

**Supplementary Table 8.** Comparison of the two best machine learning models for the classification of the time series in the groups CB-after and CW-after.

| **Parameter** | **Gaussian Process** | **Random Forest** |
| --- | --- | --- |
| Error (%) | 28.26 | 26.08 |
| Sensitivity (%) | 80.43 | 80.43 |
| Precision (%) | 77.08 | 71.15 |

**Table 9.** Results for the accuracy of all the machine learning models with standard deviation (STD) for the classification of the time series in the groups CB and CW.

| **Models** | **Accuracy (%)** | **STD (%)** |
| --- | --- | --- |
| Random Forest | 64.03 | ± 6.60 |
| SVC | 64.01 | ± 4.00 |
| Gaussian Process | 62.17 | ± 1.97 |
| DecisionTree | 61.41 | ± 2.42 |
| Linear SVC | 60.10 | ± 4.21 |
| KNeighbors | 60.10 | ± 6.23 |
| Gaussian NB | 56.75 | ± 5.95 |
| Dummy Stratified | 53.80 | ± 1.97 |

**Supplementary Table 10.** Comparison of the two best machine learning models for the classification of the time series in the groups CB and CW.

| **Parameter** | **Random Forest** | **SVC** |
| --- | --- | --- |
| Error (%) | 33.15 | 36.95 |
| Sensitivity (%) | 68.47 | 73.91 |
| Precision (%) | 66.31 | 60.71 |


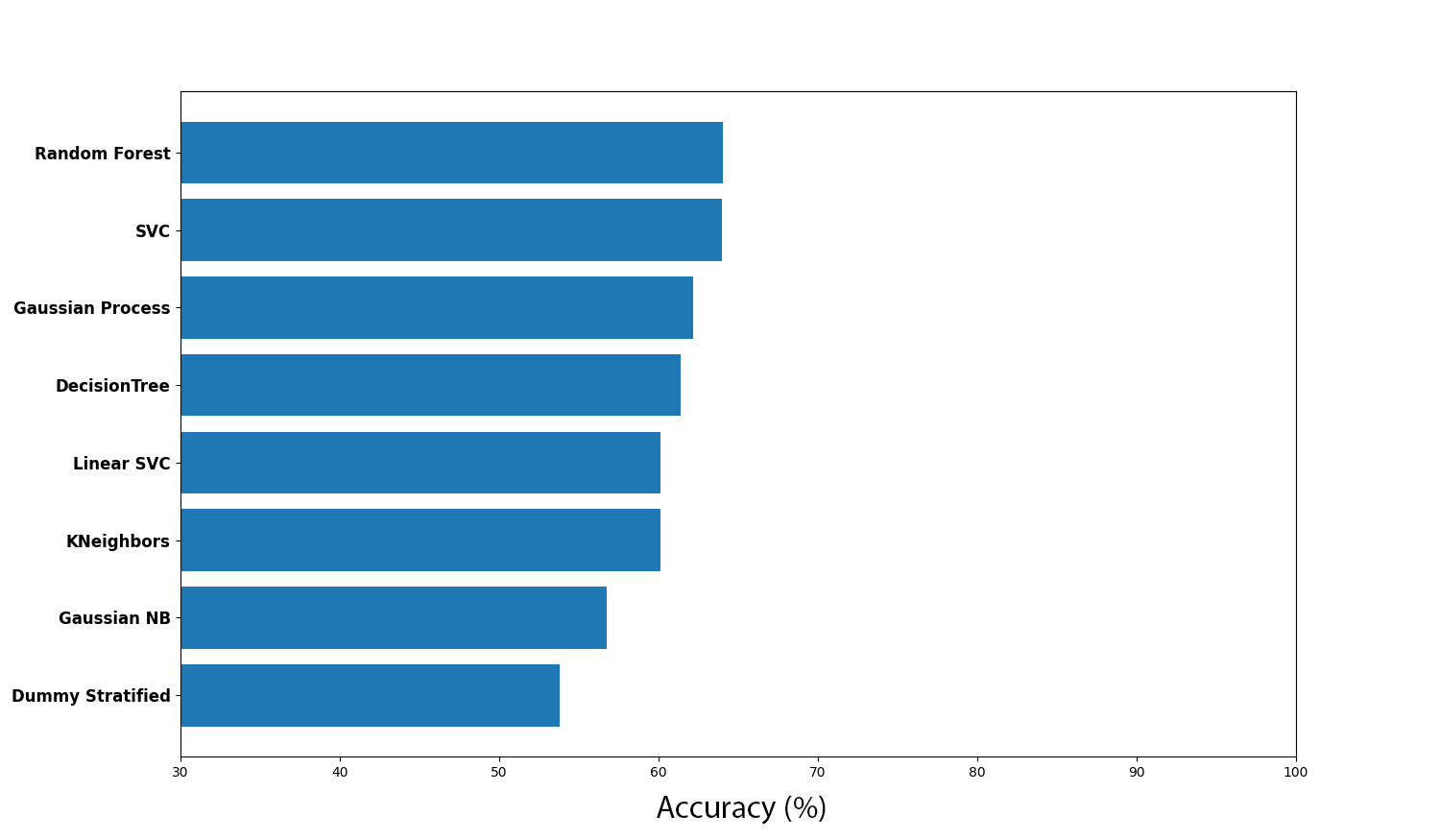


**Supplementary Figure 6.** Accuracy for the machine learning models (CB vs CW).
